# No Time for Nitrides: How Cobalt Alloying Promotes Iron Catalysts for Ammonia Decomposition

**DOI:** 10.1021/acscatal.5c04795

**Published:** 2025-09-17

**Authors:** Simone Perego, Maximilian Purcel, Yannick Baum, Shilong Chen, Astrid Sophie Müller, Michele Parrinello, Malte Behrens, Martin Muhler, Luigi Bonati

**Affiliations:** † Atomistic Simulations, 121451 Italian Institute of Technology, Genova 16163, Italy; ‡ Laboratory of Industrial Chemistry, 9142 Ruhr University Bochum, Bochum 44780, Germany; § Max Planck Institute for Chemical Energy Conversion, Mülheim an der Ruhr 45470 Germany; ∥ Institute of Inorganic Chemistry, 9179 Kiel University, Kiel 24118, Germany; ⊥ National Engineering Research Center of Chemical Fertilizer Catalyst (NERC−CFC), School of Chemical Engineering, Fuzhou University, Fuzhou 350002, China; # Kiel Nano, Surface and Interface Science KiNSIS, Kiel University, Kiel 24118, Germany

**Keywords:** ammonia decomposition, molecular dynamics, machine learning potentials, nitridation, alloying

## Abstract

The increasing demand for hydrogen production has driven interest in ammonia decomposition. Iron-based catalysts, widely used for ammonia synthesis, exhibit suboptimal performance in the reverse process due to their tendency to form iron nitrides. Recent experiments have shown that alloying iron with cobalt enhances the catalytic activity (Chen et al., *Nat. Commun.* 15, 871, 2024), yet the microscopic origin of this promotional effect is not fully understood. To address this, we leverage recent developments in machine learning-based molecular dynamics simulations to investigate the key reactions of the catalytic cycle, fully accounting for dynamical lateral interactions on the catalyst surface. Our simulations reveal that cobalt alloying provides a dual promotional effect: it slightly lowers the free energy barrier for nitrogen recombination, which is the rate-determining step for ammonia decomposition on iron, while significantly suppressing nitrogen migration into the bulk, thereby preventing nitride formation. These insights are supported by complementary transient decomposition experiments and desorption measurements, which confirm the enhanced activity and resistance to nitridation in FeCo alloys compared to monometallic iron catalysts. Furthermore, long-term stability tests demonstrate that the FeCo catalyst sustains high ammonia conversion over extended time scales. By capturing the complex interplay of competing dynamical processes at the atomic scale, our results highlight the importance of going beyond static structure–property relationships to gain mechanistic insights that can guide the rational design of more robust and efficient catalysts.

## Graphical abstract

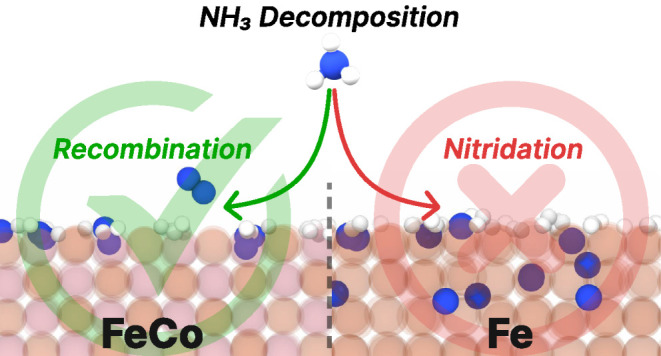

## Introduction

The urgent need to shift to a green economy has generated considerable interest in using ammonia (NH_3_) as a hydrogen carrier because of its high hydrogen content and ease of storage.^1,2^ To this end, efficient methods of extracting hydrogen must be found. Thermal decomposition of ammonia over iron-based catalysts has received much interest in recent decades, as they are already used for synthesis.^3^ However, their performance is suboptimal, and the catalysts are not stable under decomposition conditions; rather, they turn into iron nitrides.^3,4^ A sustainable way to improve them comes from making an alloy with other non-noble elements. In fact, following the idea that combining elements with different bonding characteristics can lead to optimal bonding energies for reaction intermediates^5^ several bimetallic alloys have been tested.^6–13^ In particular, here we focus on iron and cobalt alloying, for which there is multiple evidence of a promotional effect over monometallic iron.^10,12,13^ Recent work by Chen et al.^12^ reported the synthesis and characterization of bimetallic iron–cobalt catalysts supported on magnesium oxide (denoted as FeCo/MgO) derived from Mg­(Fe_0.5_Co_0.5_)_2_O_4_ spinel precursors via coprecipitation, calcination, and reduction. These catalysts, which feature high metal loadings, demonstrated enhanced catalytic activity for ammonia decomposition, with optimal performance observed at a Fe:Co ratio of 1:1. Moreover, *operando* XAS and XRD measurements revealed that the active phase corresponds to a metallic FeCo alloy with a body-centered cubic (bcc)-like structure that remains stable under reaction conditions. These findings suggest that, unlike pure Fe catalysts, the FeCo alloy resists bulk nitridation during NH_3_ decomposition. However, the origin of the improved catalytic activity over the Fe catalyst from a microscopic point of view remains elusive. In fact, the promotional effect could be ascribed to several reasons, such as changing the reaction mechanism, lowering the free energy barriers of the rate-determining steps, or preventing nitride formation.

From the theoretical side, very limited modeling has been carried out for this system, leaving us with only indirect insights. In ref. 12, the higher activity was attributed to the combination of the different N binding energies resulting in an optimal, “ruthenium-like” behavior, following the example of the CoMo alloy proposed by optimizing the volcano-like structure–property relationship.^6,14^ However, during ammonia decomposition, the cobalt molybdenum alloy formed bulk nitrides (Co_3_Mo_3_N)^15^ which does not seem to be the case for FeCo alloys, leaving us with an incomplete picture. In ref. 13, while experiments were performed on Fe–Co alloys, the computational analysis was limited to pure Fe and Co systems. The authors attributed the improved FeCo alloy activity to the presence of Co and suggested that the reaction mechanism would resemble an ideal average between the behaviors of the two monometallic catalysts. In particular, they indicated that the free energy barriers would likely resemble those of Co, but with a reduction in the hydrogen recombination barrier, which is considered limiting on monometallic Co.

Only recently, thanks to the combination of machine learning-based interatomic potentials^16–20^ and enhanced sampling techniques^21–23^ dynamical simulations have become feasible tools to investigate catalytic processes under realistic conditions.^24–32^ Using these methodologies, it has been possible to simulate ammonia synthesis^26,28^ and decomposition^29,33^ over iron catalysts, providing atomistic insights into the role of dynamics under operando conditions. Furthermore, we recently developed a data-efficient approach to construct accurate machine learning potentials with minimal training data^34^ initially demonstrated on selected elementary steps as a methodological proof of concept. Building on these advancements, we are now able to investigate the key reactions of the catalytic cycle under more realistic conditions. In particular, we aim to go beyond conventional static approaches by modeling adsorbate lateral interactions in a fully dynamical manner. These interactions can indeed have pronounced effects, such as modifying free energy barriers and inducing local surface restructuring.^28,35,36^

In this paper, we present a comprehensive investigation into the catalytic behavior of FeCo alloys during ammonia decomposition and nitrogen release, combining advanced MD simulations and experiments. Since the activity and structure properties of the catalyst have already been characterized experimentally^12^ we begin by presenting theoretical insights aimed at uncovering the atomistic origin of these observed properties. The same order has also been adopted in the Methods sections and Supporting Information. The simulations focus on nitrogen recombination and migration within the material, indeed the former being the rate-determining step on Fe catalysts, and the latter playing a central role in nitride formation.^33^ We find that alloying iron with cobalt imparts a multiple promotional effect: while it (slightly) reduces the free energy barrier for the rate-limiting nitrogen recombination, it also suppresses nitrogen migration into the bulk, thereby preventing nitride formation. These findings are validated by complementary experiments using FeCo/MgO catalysts synthesized via coprecipitation and reduction of spinel precursors, as described in ref. 12. Here, to characterize the potential nitridation of the catalyst, we perform transient and steady-state NH_3_ decomposition experiments as well as temperature-programmed desorption (TPD) measurements. The long-term performance of the catalyst over extended time scales (1000 h) is also reported. These experiments confirm the higher activity and stability against nitridation of Fe–Co alloys under ammonia decomposition conditions with respect to the iron-based one. Together, our combined theoretical and experimental approach provides atomistic insights into the origin of catalytic promotion in FeCo alloys and offers guiding principles for the rational design of more effective catalysts.

## Results

### Data-Efficient Construction of Machine Learning Interatomic Potential

The first step in the dynamical modeling of the catalytic process is the construction of an accurate interatomic potential. To this end, we employed the Data-Efficient Active Learning (DEAL) scheme introduced in ref. 34 to iteratively build a machine learning (ML) potential capable of describing nitrogen recombination and migration on FeCo(110) under realistic conditions. These include finite surface coverage of both N and H to account for lateral interactions (see Methods).

Starting from an initial machine learning potential trained to model NH_3_ dehydrogenation on a clean surface^34^ we significantly extend its applicability to (1) include nitrogen recombination and dissolution reactions, and (2) capture behavior across a range of surface coverages. In each iteration of the active learning loop (see Figure S1), the targeted reactions were simulated using the current ML potential. Then we used DEAL to select a minimal subset of configurations, which were recalculated at the DFT level and added to the training data set. The procedure was repeated until the model’s uncertainty fell below the predefined threshold across all coverages (Figures S2 and S3), resulting in a compact training set of approximately 3,000 DFT configurations (Figure 1 inset and Table S1), which contrasts with the over 200,000 configurations used in previous studies of lateral interactions on Fe.^28^

**Figure 1:**
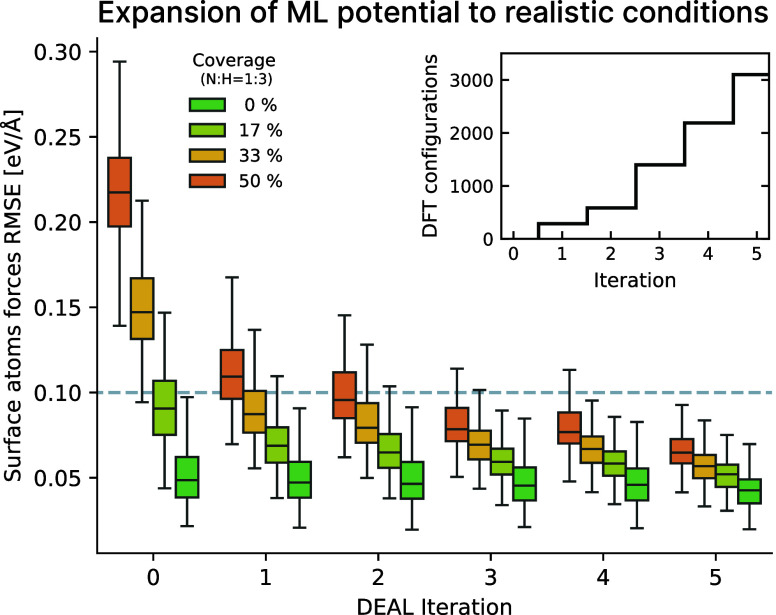
Evolution of the accuracy of the ML potential during iterative expansion with DEAL. Boxplot of the root-mean-square error (RMSE) of forces on surface atoms (top FeCo layer and adsorbates) for different ML potentials, evaluated on an independent test data set uniformly distributed along the reaction path (see Methods). The results are shown for different surface coverages. In the inset, the cumulative number of DFT configurations added to the starting data set is reported.

The effectiveness of this approach was validated using an independent test set, which also enabled us to track the progressive improvement across active learning iterations. The final ML potential reaches a mean absolute error of 0.31 meV/atom in energies and 12 meV/Å in forces. Given the heterogeneity of the system, however, it is more informative to examine the force accuracy on reactants and surface atoms, where errors are substantially larger (Figure 1). Our procedure systematically reduces these errors below 0.1 eV/Å (gray line) across all coverages. In addition, the model reaches a uniform accuracy along the reaction pathways, a crucial requirement for a data-driven description of reactive events (Figures S1 and S2). In the Supporting Information, we also report an additional validation of the long-term reliability by recalculating DFT energies and forces of configurations extracted from nanosecond-long reactive simulations (Figure S4). Altogether, this extended validation demonstrates the robustness of our approach for simulating relevant reactions under finite-coverage conditions.

### The Rate-Limiting Step: Nitrogen Recombination

Having constructed the ML potential, we proceeded to study nitrogen recombination at the operative temperature of 700 K, where ammonia conversion is observed experimentally. We began by analyzing the diffusion of atomic nitrogen adsorbed on the surface (N*). The free energy surface (FES) projected along the two in-plane crystallographic directions reveals that it is preferentially adsorbed at the hollow sites (*h*) coordinated with Fe rather than Co atoms (Figure S5). The diffusion mechanism passes through a short bridge site with a barrier of ∼ 75 kJ mol^–1^, similar to what has been found for Fe surfaces.^33^ By studying the behavior of diffusion in proximity to another N, we identify the configuration in which two N* are in adjacent hollow sites to be the precursor of the N–N recombination (Figure S5). To investigate the nitrogen recombinative desorption, we performed enhanced sampling simulations starting from this state, which allowed us to calculate the free energy profile and characterize its mechanism in detail.

Since charge transfer is known to play a key role in the adsorption and dissociation of N_2_ ^37,38^ we followed the strategy of ref. 26 and included partial atomic charges as an additional learned property of the ML model (see Methods). This enables the prediction of charge transfer without requiring additional DFT calculations. Notably, the free energy surface computed as a function of the cumulative charge on the two N atoms, *q*(N_2_), provides an electronic perspective on the reaction mechanism while fully accounting for temperature effects (Figure 2).

**Figure 2:**
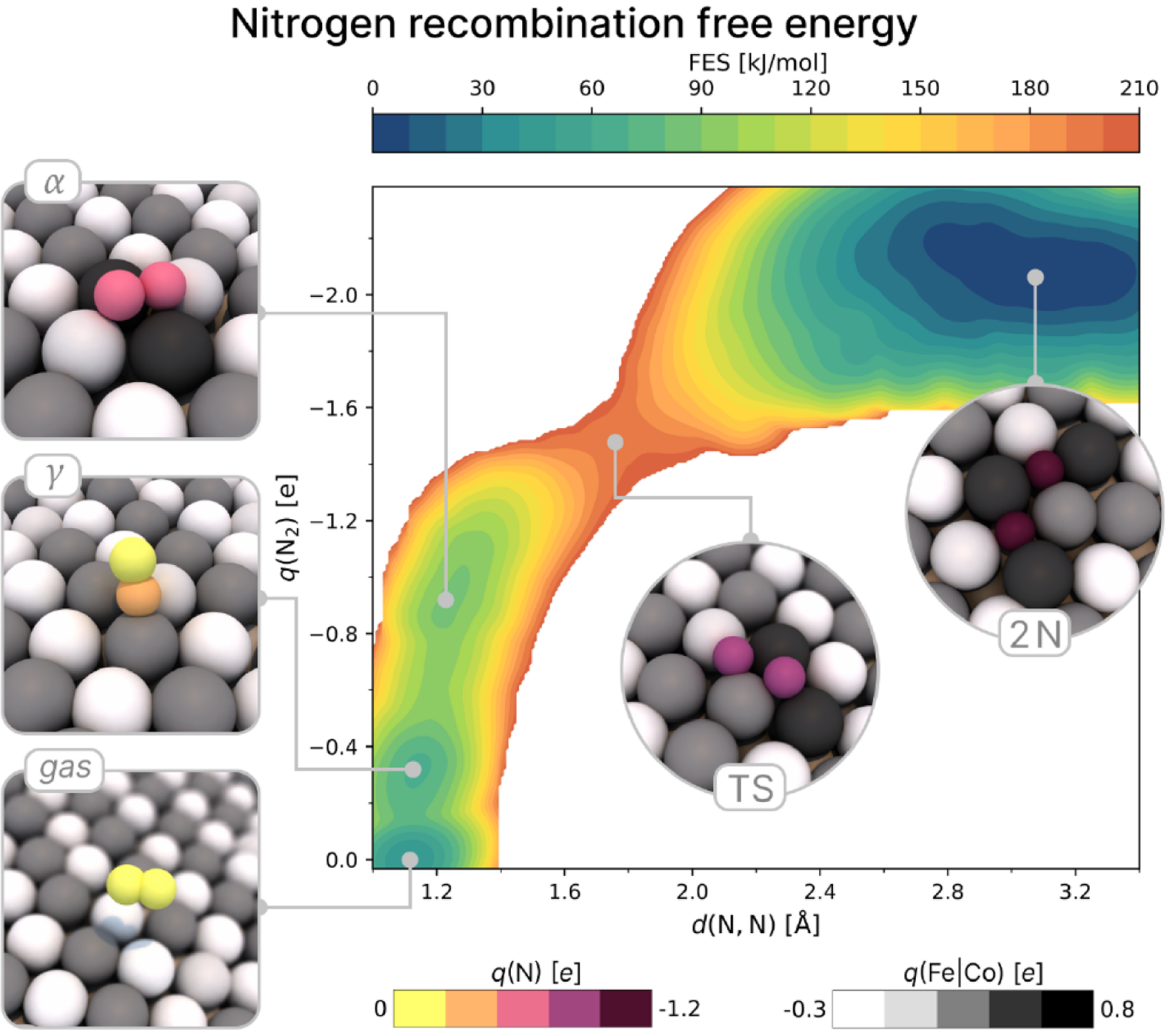
Free energy surface (FES) of nitrogen recombination/dissociation (2N* ⇌ N_2_) as a function of N–N distance *d*(N,N) and the total charge transferred from the metal catalyst to the nitrogen atoms *q*(N_2_). The local minima on the plots correspond to metastable states. Some snapshots along the reaction are reported. From right to left: reactants (2N), transition state (TS), various N_2_ adsorption sites (α, and γ) and products (N_2_ gas phase). The atoms are colored according to their partial charge predicted by the ML model. The FES of the same process as a function of *d*(N,N) and the coordination between nitrogen and the metal atoms *C*_N,Fe|Co_ is reported in Figure S6.

By inspecting the free energy profile, we can identify the key intermediates of the process. Starting from the precursor state in which two N* atoms are adsorbed at adjacent *h* sites (2N), the reaction occurs in the connecting short bridge site, and upon recombination and formation of the triple bond, the charge transfer *q*(N_2_) decreases significantly. The resulting N_2_ molecule is initially adsorbed in a horizontal configuration above long bridges (α), before relaxing into a vertical site (γ) from which it can desorb. During this process, the charge transfer is progressively reduced, resulting in a weakening of the metal–adsorbate interaction, which eventually leads to the N_2_ release.

The computed free energy barrier for N–N recombination is approximately 190 kJ mol^–1^, which is substantially higher than the barriers for the dehydrogenation steps (106, 77, and 127 kJ mol^–1^ for NH_3_ → NH_2_ + H, NH_2_ → NH + H, and NH → N + H, respectively) calculated in ref. 34. This confirms that N–N recombination is the rate-determining step for ammonia decomposition on this alloy. Alloying with Co lowers the barrier compared to pure Fe (216 kJ mol^–1^)^33^ but the reduction is moderate and the value remains well above that of pure Co surfaces (120 kJ mol^–1^).^13^ This finding challenges the hypothesis that the alloy inherits the catalytic and kinetic properties of Co.^13^

### Alloying Makes Nitridation Unfavorable

While the free energy barrier for recombination is a key quantity, it must be considered alongside a competing process: nitrogen migration into the subsurface and eventually the bulk. This process is indeed the initial step toward the formation of bulk nitrides. In ref. 33, we showed that for pure Fe, migration into the bulk is favored over recombination due to a lower barrier, leading to the progressive nitridation of the material during ammonia decomposition. To investigate whether a similar mechanism is operative on the FeCo alloy, we reconstructed the free energy profile of nitrogen dissolution and segregation.

The migration pathway is described using the variable *v*_01̅0_, which tracks the position of the nitrogen atom along the crystallographic direction [01̅0] (Figure 3). Starting from a hollow adsorption site on the surface, nitrogen first migrates to a nearby octahedral interstitial site, which corresponds to the small shoulder at *v*_01̅0_ ≈ 1, and then proceeds to a second octahedral site that constitutes the first metastable configuration. From there, nitrogen alternates between two distinct interstitial sites of the CsCl-like bulk structure, forming either two short bonds with Co and four longer ones with Fe, or vice versa (see snapshots in Figure 3. The configuration favoring shorter bonds with Fe is more stable, indicating a weaker affinity between nitrogen and cobalt.

**Figure 3:**
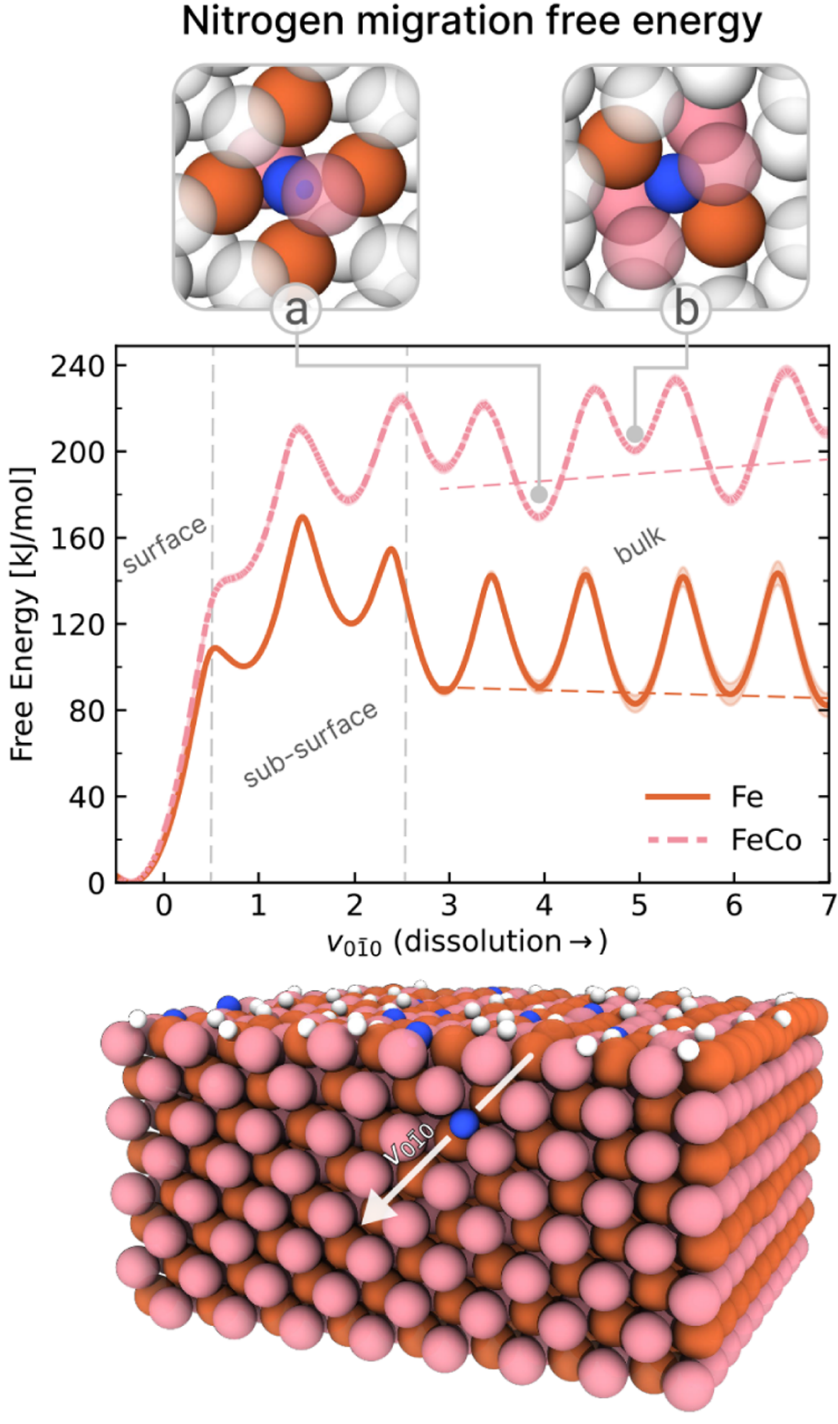
Free energy profiles of nitrogen dissolution/segregation on Fe(110) and FeCo(110) slabs at *T* = 700 K. The free energy is projected along the *v*_01̅0_ direction. The Fe data are reproduced from ref. 33. In the insets, we report two snapshots of the different FeCo octahedral interstitial where N is placed at a) the center of the face of the Fe simple cubic lattice and b) at the center of the face of the Co simple cubic lattice. The uncertainty of the free energy profiles is computed as the standard deviation of four independent simulations performed with four different ML potentials. Additionally, to highlight the trend of the free energy profile as the dissolution inside the bulk proceeds, we report the linear interpolation of the metastable free energy minima with dashed lines (*v*_01̅0_ > 2.5). In the bottom, we report a frame from a large-scale simulation of FeCo(110) slab at 40% of monolayer coverage, where the [0 1̅ 0] diffusion “channel” is highlighted. The free energy profiles at finite coverage are reported in Figure S7.

Comparing the overall barrier for migration from surface to bulk (*v*_01̅0_ ≥ 2.5) between the two catalysts, FeCo exhibits a significantly higher barrier (223 kJ mol^–1^) than Fe (169 kJ mol^–1^). Additionally, we observe that the free energy barriers for dissolution are slightly higher (by ∼ 5 kJ mol^–1^) than for segregation, which is reflected in the increasing energy of the metastable minima as nitrogen moves deeper into the bulk (pink dotted line). This trend suggests that even when nitrogen penetrates below the surface layers of FeCo, the likelihood of continued migration is lower than that of the reverse process. This behavior stands in sharp contrast to what is observed for Fe (orange solid line), where subsurface incorporation is favored.

Finally, the lower affinity of FeCo for bulk nitrogen is also evident from the free energy difference between adsorbed and dissolved states. At the interstitial site located in the third layer (*v*_01̅0_ ≈ 4), this difference is approximately 80 kJ mol^–1^ higher for FeCo than for Fe. Taken together, these results indicate that not only is nitrogen incorporation into the bulk of FeCo kinetically hindered, but its thermodynamic stability within the bulk is also reduced, leading to a suppression of the nitridation process in the alloy.

To further investigate the origin of the different interactions between the two catalysts, we analyzed the amount of charge transferred to nitrogen during the migration process (see Table S3). We find that pure Fe, as well as Fe-rich sites in FeCo, transfer more charge to nitrogen compared to Co-rich sites. This indicates a weakening of the catalyst-N interaction in the alloy, consistent with the increased dissolution barrier. At the same time, the weaker surface interaction also contributes to the reduced N–N recombination barrier, since weaker binding to the surface facilitates interaction between adsorbed nitrogen atoms.

### The Effects of Lateral Interactions on Nitrogen Release

Having characterized the two elementary steps, N recombination and migration, we now increase the complexity of our analysis by introducing lateral interactions arising from finite surface coverage. This is a critical component for realistic modeling of catalytic surfaces, yet it has often been treated only approximately, for example via static calculations on small supercells, which can yield unphysical adsorbate concentrations. Thanks to the development of data-efficient machine learning potentials trained to accurately describe a wide range of reaction environments, we are now able to simulate these interactions in a full dynamical framework.

To facilitate comparison with Fe, we adopted the same setup as in ref. 33 running simulations at 700 K with finite coverages of N and H atoms (20% and 40% of a monolayer, respectively, maintaining an N:H ratio of 1:3). From molecular dynamics simulations, we computed the free energy profiles for both recombination and migration as functions of surface coverage (Figure S7). For recombination, we focused on the barrier from the precursor configuration, which is the rate-determining step. As coverage increases, both recombination and migration barriers decrease. In particular, the barrier for entry into the first octahedral interstitial site at *v*_01̅0_ ≈ 1 is lowered, and the site becomes more stable with increasing coverage. These trends are summarized in Figure 4 for both FeCo (left) and Fe (right).

**Figure 4:**
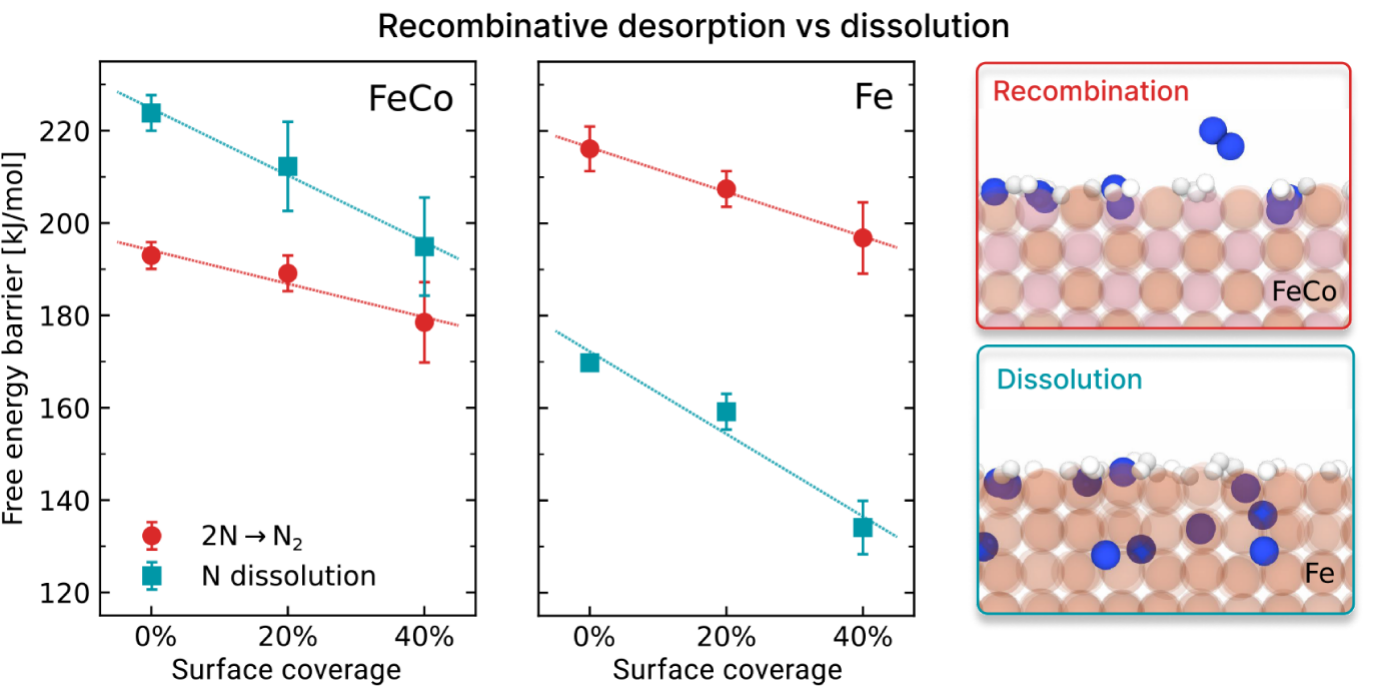
Coverage-dependent free energy barriers for both N recombination (red) and migration inside the bulk (blue) on Fe^33^ (left) and FeCo alloy (right) surfaces. The surface coverage refers to N, H monolayer coverage with an N:H ratio equal to 1:3. The Fe barriers are reproduced from ref. 33, while for FeCo are extracted from Figure S7. Dashed lines are obtained from linear regression. The snapshots show a visual representation of the most likely process on each of the two catalysts.

Although both recombination and migration barriers decrease with increasing coverage, nitrogen release via recombination remains the more favorable pathway on FeCo. While Fe consistently favors migration, which accounts for the progressive nitridation of the catalyst as nitrogen tends to penetrate and remain in the bulk, the opposite behavior is observed on FeCo. Even if recombination and migration barriers may become comparable near full monolayer coverage, our previous analysis showed that nitrogen is much less likely to remain in the bulk once it migrates below the surface. As a result, even in cases of subsurface incorporation, the probability of long-term retention is low, effectively suppressing bulk nitridation.

### Experimental Observation of Nitridation Prevention via Cobalt Alloying

To validate the picture originating from the simulations, and particularly the effect of alloying on the competition between the migration and recombination for nitrogen release, we investigated the nitride formation during ammonia decomposition on the spinel-derived Fe and FeCo catalysts supported on magnesium oxide (denoted as Fe/MgO and FeCo/MgO for brevity), prepared as in ref. 12. The characterization and prior reduction of the catalysts are reported in the SI.

We first perform transient NH_3_ decomposition, where the reduced catalysts were heated in a flow of 1.33% NH_3_/He (100 mL_n_ min^–1^, both 99.999%) to 873 K with a heating rate of 1 K min^–1^. The maximum temperature was held for 1 h before cooling to 423 K at the same rate as before. This transient NH_3_ decomposition cycle was repeated a second time for each sample to assess any changes in the behavior due to reaction-induced transformations of the catalysts. The effluent mole fractions of N_2_, H_2_, and NH_3_ during the first and second cycles are shown in Figure 5 for the two catalysts. For the Fe catalyst, during the first cycle (Figure 5a), a shoulder in the H_2_ release curve can be observed at about 600 K (327 °C). This behavior is marked by a decrease in the ammonia fraction and a corresponding increase in H_2_, without the simultaneous release of N_2_, indicating nitrogen incorporation into the bulk and subsequent formation of iron nitrides.^33^ This can be rationalized in light of simulations showing that dehydrogenation steps have lower barriers^29^ than nitrogen recombination and that N dissolution is favored over recombination.^33^ Indeed, the same behavior had been observed on a wustite-based Fe catalyst.^33^ During the second cycle of NH_3_ transient decomposition (Figure 5b), no shoulder in the H_2_ release is observed around 600 K, but only a release of H_2_ and N_2_ in a stoichiometric ratio of 3:1. The absence of nitride formation in the second cycle can be attributed to the fact that bulk nitrides are already present, indicating that nitride formation/decomposition is reversible (nitrides reform during the cooling procedure after the first cycle), similar to what was observed in the case of wustite-based bulk iron catalysts.^33^ In contrast, at high temperatures, the behavior is identical to that of the first cycle for the release of N_2_, showing a peak around 800 K associated with the decomposition of nitrides. It is worth noting that, at the same time as N_2_ release, we observe a sharp increase in NH_3_ conversion. This suggests that pure Fe has greater activity than its nitride phase, or in other words, that nitrogen poisons the catalyst below such a temperature.

**Figure 5:**
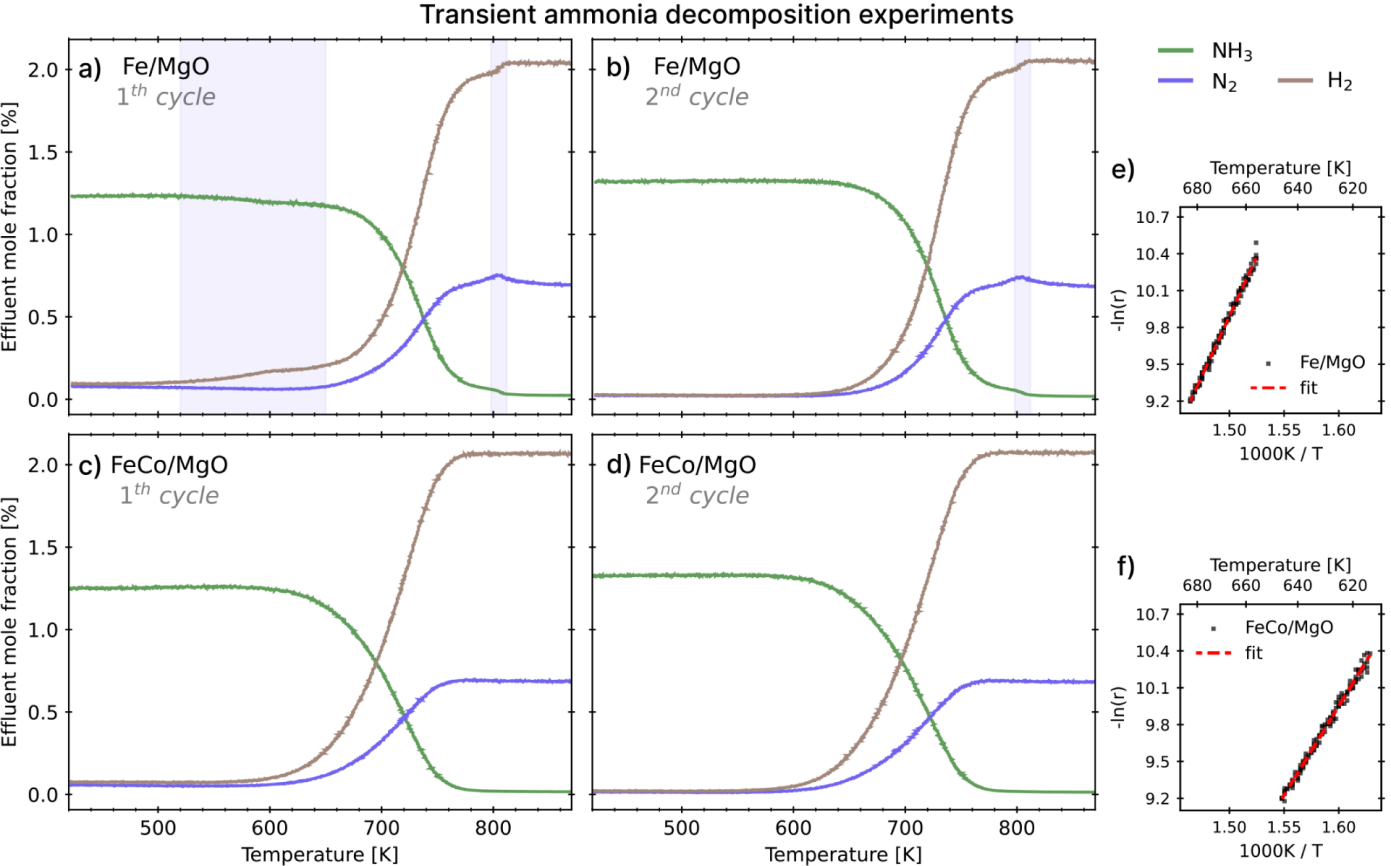
First and second cycles of transient NH_3_ decomposition using (a,b) Fe/MgO and (c,d) FeCo/MgO (1.33% NH_3_ in He, 423 K-873 K (150 °C-600 °C), β = 1 K min^–1^). The catalysts were reduced before the experiments. The blue-shaded area reported for Fe/MgO highlights the temperature range where nitride formation/decomposition occurs (see text). In the insets on the right, we report the Arrhenius plots used to calculate the apparent activation energy for NH_3_ decomposition over (e) Fe/MgO and (f) FeCo/MgO. Data are extracted from the second cycle of transient NH_3_ decomposition up to 10% conversion.

In contrast, the Co-containing spinel exhibits identical behavior in the first (Figure 5c) and second cycles (Figure 5d) of transient NH_3_ decomposition. For FeCo/MgO, the absence of a low-temperature shoulder in the H_2_ signal during the first cycle indicates suppression of nitrogen incorporation, in agreement with simulation predictions. In the alloy, atomic nitrogen dissolution is an activated process with a higher barrier than recombinative desorption, opposite to what is observed for Fe. Moreover, the N_2_ signal at elevated temperatures further confirms the absence of nitride decomposition, as no anomalous increase is detected.

From the transient decomposition experiments, we also derived the apparent activation energies for NH_3_ decomposition over Fe/MgO and FeCo/MgO catalysts, based on the Arrhenius plot obtained from the second cycle of transient NH_3_ decomposition, evaluated up to 10% conversion (Figure 5e,f). The partial substitution of Fe with Co in the spinel significantly reduces the apparent activation energy, from ∼ 160 kJ mol^–1^ to ∼ 120 kJ mol^–1^.

Furthermore, we conducted complementary steady-state NH_3_ decomposition measurements between 673 and 873 K (see Methods). From these measurements we extract a similar decrease in activation energy from ∼ 150 kJ mol^–1^ to ∼ 104 kJ mol^–1^ (Figure S11) which is particularly remarkable given the high sensitivity on the temperature and conversion range of the exponential fitting. Interestingly, the results for the FeCo/MgO catalyst closely match those of previously reported samples^12^ while the Fe-only catalyst shows greater variability in activation energy, although its overall catalytic activity is comparable to that of the previously reported Fe-only samples^12^ (Figure S12). This might be attributed to differences in the material’s properties in both the precursor and the iron nitride phases (Figure S8). Such variability is characteristic of the pure iron system, which can form a range of nitrided phases under reaction conditions.^33^ In contrast, the bimetallic alloy exhibits more stable structural and catalytic properties, as supported by atomistic simulations and by SEM images (Figure S9) and EDX maps (Figure S10) of the spent FeCo/MgO catalyst, confirming that a uniform porous morphology and a homogeneous element distribution are maintained after reaction.

### Desorption after Transient NH_3_ Decomposition: No Bulk Nitride Formation

To further investigate the different behavior with respect to nitridation of the two catalysts, we compare the desorption profiles after transient NH_3_ decomposition and 30 min NH_3_ exposure at 423 K (Figures 6a and S13). The desorption of N_2_ from Fe/MgO (Figure 6a) is a further indication of the formation of nitrides during transient NH_3_ decomposition. Integration yields an amount of 3.78 mmol $g_{\mathrm{cat}}^{-1}$ of atomic N that was taken up by the catalyst. The overall N/Fe ratio was calculated to be 0.38, which corresponds to a Fe:N stoichiometry of the nitride between 2:1 and 3:1, indicating the formation of bulk nitrides. The desorption profile of N_2_ shows a similar shape and similar characteristics as the desorption of N_2_ from promoted Fe bulk catalyst, where a two-step nitride formation and decomposition has been found.^33^ For FeCo/MgO the amount of stored atomic N was approximately 1 order of magnitude lower with 0.37 mmol $g_{\mathrm{cat}}^{-1}$ and only one desorption maximum at 640 K (367 °C) was found. The substitution of Co into the spinel, which lowers the Fe content by 50%, decreased the amount of atomic N desorbed from the catalyst after transient NH_3_ decomposition by 90%. The reduced amount of N_2_ points toward a surface coverage of FeCo/MgO with chemisorbed N instead of bulk nitride formation. Finally, the desorption of unreacted NH_3_ is similar for both catalysts, and only minor amounts of NH_3_ were detected (Figure S13).

**Figure 6:**
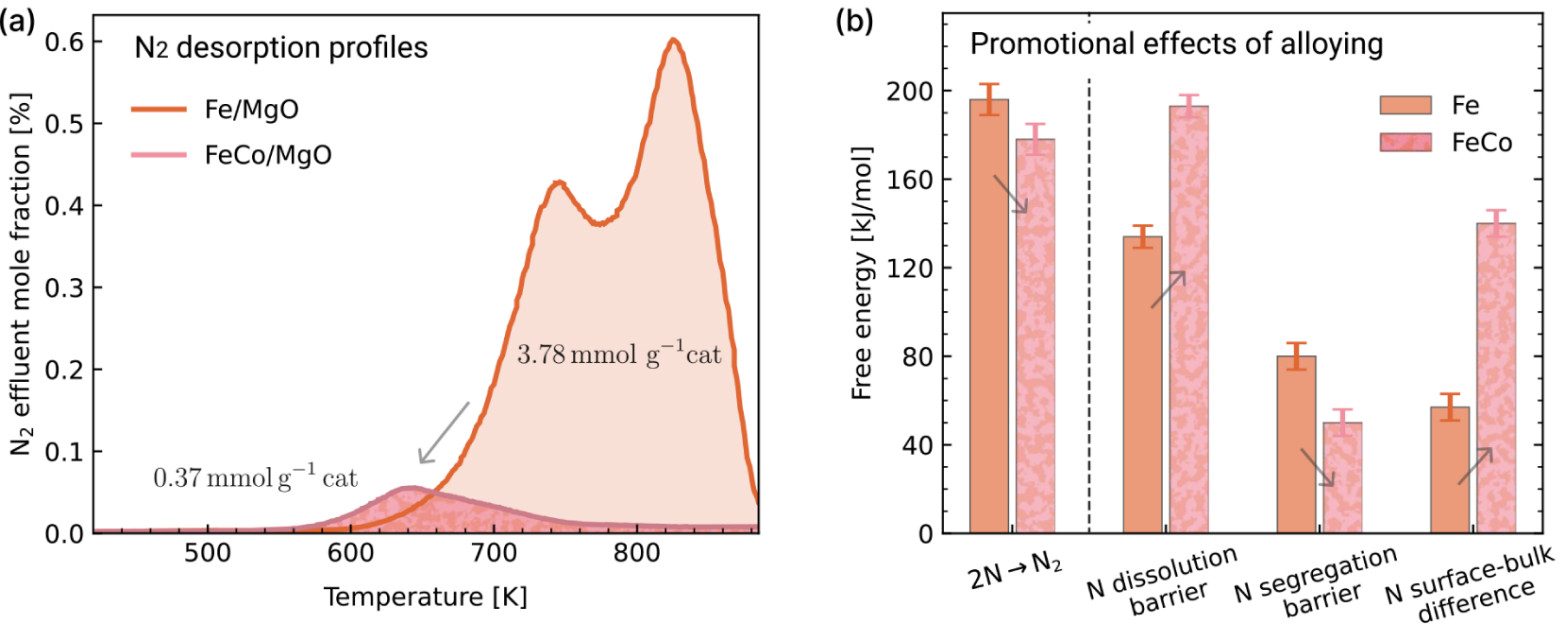
(a) Temperature-programmed desorption (TPD) profiles of N_2_ from spent Fe/MgO and FeCo/MgO catalysts following 30 min NH_3_ exposure at 423 K (pure He, 423 K-873 K, β = 5 K min^–1^). The total amount of atomic nitrogen desorbed as N_2_ during the TPD experiment is equivalent to 3.78 mmol $g_{\mathrm{cat}}^{-1}$ for Fe/MgO and 0.37 mmol $g_{\mathrm{cat}}^{-1}$ for FeCo/MgO, and reported in the figure. (b) Summary of the computational results for the most relevant steps in NH_3_ decomposition on Fe and FeCo catalysts. The reported free energies correspond to simulations under high surface coverage conditions (40% of a monolayer, with an N:H ratio of 1:3), and are obtained from fully dynamical simulations. Arrows highlight the multiple promotional effects induced by Co alloying in Fe, which explain the experimentally observed improvements in activity and resistance to nitridation.

### Long-Term Stability Measurements

Finally, to assess the durability of the FeCo/MgO catalyst under prolonged reaction conditions, we conducted long-term ammonia decomposition experiments, which are reported in Figure S14. At 558 °C (831 K, measured inside the catalyst bed) and using a feed of 98% NH_3_ in Ar, the alloy catalyst maintained a high ammonia conversion over 1000 h on stream. After a brief initial activation, during which the conversion increased from 88% by about 1% the catalyst exhibited a slow and steady deactivation rate of only ∼ 0.6% per 100 h. This stability is comparable to Ni- and Ru-based catalysts reported in the literature^39^ and only slightly lower than that of promoted industrial Ni catalysts, which show deactivation rates of ∼ 0.2% per 100 h under comparable conditions.^40^ These results demonstrate that cobalt alloying not only suppresses nitride formation during transient operation, but also enables sustained high activity over extended time scales, validating its role as an effective stabilizing strategy against nitridation.

## Conclusion

The primary objective of this study was to understand the origin of the enhanced catalytic activity observed experimentally for FeCo alloy catalysts during ammonia decomposition.^12^ Prior literature has proposed that this improvement could stem from cobalt-like catalytic behavior, involving significant changes in reaction free energies, or alternatively, from the suppression of catalyst nitridation. However, achieving an atomistic understanding of these mechanisms under *operando* conditions remains extremely challenging using experimental methods alone. To bridge this gap, we employed *ab initio*-quality molecular dynamics simulations, enabled by a highly data-efficient machine learning potential capable of accurately describing all relevant reaction steps on the FeCo alloy. Leveraging a recent active learning approach^34^ we constructed this potential using only a few thousand DFT calculations, which is 2 orders of magnitude fewer than those required in earlier studies.^29^ Crucially, this allowed us to simulate realistic environments with finite surface coverage and lateral interactions in a fully dynamic fashion, avoiding the limitations of static approximations.

The resulting long-time scale simulations yielded key mechanistic insights into the promotional role of Co alloying. As summarized in Figure 6b, we found that alloying slightly lowers the free energy barrier for N–N surface recombination, which is the rate-determining step on both Fe and FeCo surfaces. However, the decrease is far less pronounced than previously hypothesized, and the reaction mechanism remains much closer to that of Fe, which shares the same bcc structure, confirming the high structure sensitivity of these reactions.^41^ Notably, our simulations revealed a significant increase in the free energy barrier for nitrogen migration into the bulk, fundamentally altering the balance between recombination and migration. While Fe favors migration, facilitating nitridation via subsurface incorporation, FeCo favors recombination, promoting nitrogen release from the surface and suppressing nitridation. Another key finding is the thermodynamic destabilization of bulk nitrides in FeCo alloys. Nitrogen atoms in the bulk are less stable, and the barrier for segregation back to the surface is lower than in pure Fe. These resistance effects are intrinsic to the alloyed bulk structure and cannot be attributed to individual cobalt atoms.

The experimental investigations performed in this study complement both the advanced simulations and the previously reported structural and chemical characterization of FeCo/MgO alloys.^12^ Transient ammonia decomposition and N_2_ desorption measurements revealed enhanced catalytic activity and a marked resistance to nitride formation compared to Fe/MgO catalysts. These findings are further supported by long-term stability measurements, which demonstrate that the FeCo catalyst sustains high ammonia conversion over 1000 h with minimal deactivation. Together, these results validate the mechanistic insights from simulations and highlight how cobalt alloying impacts both the kinetics and stability of the catalytic system. In summary, the promotional effect of cobalt alloying is multifaceted and arises from two complementary mechanisms: (i) a moderate reduction of the recombination barrier, and (ii) a more profound resistance to nitridation, achieved by suppressing nitrogen migration and destabilizing bulk-incorporated species.

These findings have broader implications beyond the specific system studied. Our work highlights the intricate interplay of surface and bulk properties in determining catalytic performance, challenging the view that catalytic activity can be captured by simple surrogate descriptors. Understanding such mechanisms not only deepens our fundamental knowledge of alloy catalysis but also informs rational catalyst design strategies. For instance, promoters or cocatalysts could be used to selectively optimize either recombination or migration pathways. Through the combination of advanced simulation techniques and experimental validation, this study offers a refined perspective on the promotional effects of alloying and demonstrates how integrated approaches can advance the rational design of catalytic materials.

## Computational Methods

### Simulation Setup

Following the experimental characterization (Figure S8), we modeled the catalytic system using a semi-infinite slab geometry based on the body-centered cubic (bcc) FeCo alloy in the CsCl-type structure, using a lattice parameter equal to 2.87 Å, exposing the (110) facet. Given the relatively large size of the nanoparticles (Table S4), the (110) surface is indeed expected to be the most exposed facet, as it is the thermodynamically most stable.

For the construction of the training data set of DFT calculations, we simulated FeCo slabs cut along the (110) surface containing 120 atoms (4 × 6 × 5) and 144 (4 × 6 × 6) atoms, for recombination and dissolution/segregation, respectively. A vacuum region of at least 12 Å, was added in all configurations to avoid self-interaction effects. The slab was simulated in a bulk-terminated configuration by fixing the two bottom layers. Periodic boundary conditions were applied in the x and y directions, while a reflecting wall was implemented at 10 Å over the surface in the *z*-direction to avoid N_2_ escape.

In the production simulations model, to model the catalytic environment, we used a FeCo(110) slab composed of 8 layers and 320 atoms, with the bottom two layers fixed to reproduce a bulk-terminated surface. A finite coverage of N and H atoms of 0, 20 and 40% monolayer with an N:H ratio of 1:3 was simulated to account for lateral interactions. This setup was chosen to match that used in ref. 33 for Fe, to enable direct comparison. To rule out finite-size effects, we also repeated the simulations with larger systems up to ∼ 1350 atoms.

### Machine Learning Potential

To construct the interatomic potential for modeling nitrogen recombination and migration on FeCo(110) under realistic conditions, we employed the equivariant graph neural network MACE (Message-Passing Atomic Cluster Expansion) architecture^19^ together with the Data-Efficient Active Learning (DEAL) scheme.^34^ We extended the training set from ref. 34 , initially developed for modeling only ammonia dehydrogenation on clean surfaces of FeCo, to include configurations relevant to nitrogen recombination and migration in the presence of coadsorbed species. Following an iterative active learning strategy (Figure S1), we progressively incorporated surface coverage effects by simulating recombination and migration at various nitrogen and hydrogen coverages, including up to 50% (only N, only H, and N:H ratios of 1:1 and 1:3). The first two iterations involved standard molecular dynamics, while in the remaining three we performed enhanced sampling simulations (see below). At each iteration, DEAL selected configurations in two steps: first, we evaluated the uncertainty of the force predictions of the machine learning potential, using the query-by-committee method^42^ which uses an ensemble of ML potentials. This allowed us to identify a set of configurations not properly described by the current generation. Then, a sparse Gaussian Process (GP)^43^ was trained on these preselected configurations to identify a nonredundant subset of structurally diverse samples. This ensured uniform accuracy across the reaction space while minimizing the computational cost. For the GP, local environments were described using ACE B2 descriptors^44^ with basis expansion parameters *N*_max_ = 8 and *l*_max_ = 3, and cutoff distances of 5.5 Å for Fe and Co, and 4 Å for interactions involving other species. The kernel used for comparing atomic environments was a squared normalized dot product.

The active learning cycle was repeated for five iterations until the uncertainty distribution fell consistently below the target threshold (maximum force deviation $\sigma_{\max}^{\left(\mathrm{MACE}\right)}>90$ meV/Å), enabling the ML potential to capture lateral interaction effects with high fidelity. This threshold was set in line with previous studies without lateral interactions, where it was determined by analyzing the distribution of force uncertainties.^34^ However, since uncertainty is not equivalent to the actual error with respect to DFT, we assessed the final model’s accuracy on an independent test data set composed of configurations uniformly distributed along the reaction paths. Further details on the data set and active learning protocol are provided in the Supporting Information.

All MACE models used a 6 Å cutoff radius, 256 channels, and angular momentum *L* = 0, offering a good trade-off between accuracy and computational cost. The data set was split into training and validation sets with a 95:5 ratio. Models were optimized using AMSGrad^45^ with a learning rate of 0.001 and a batch size of 20. A weighted root-mean-square error (RMSE) loss was used, with force and energy weights set to 100 and 1, respectively. In the final ∼ 25% of epochs, the energy weight was increased by a factor of 1000, while the learning rate was reduced to 0.0001. Training was halted using early stopping with a patience of 200 epochs.

Besides learning energy and forces, we also train the MACE model to learn the atomic charges, whose labels are obtained from DFT calculations via the Bader decomposition scheme.^46,47^ To this end, we included an extra term in the loss function for the atomic charge, with a weight of 0.1 in the first part of the training and raised to 100 in the second stage. A weight of 0 was set to configurations featuring an unphysical value of the partial charge arising from the misassignment of valence electrons in the Bader partitioning scheme. This results in a mean absolute error of 0.004 *e*.

### Molecular Dynamics

MD simulations were performed using the Large-scale Atomic/Molecular Massively Parallel Simulator (LAMMPS) software^48^ together with MACE v0.3.0 ^19^ and PLUMED v2.9.^49^ The canonical sampling through velocity rescaling thermostat^50^ with a coupling constant of 50 fs was employed to control the temperature in all the simulations, and the integration time step was set to 0.5 fs.

To assign the uncertainty to the free energy profiles extracted from the MD simulations, we performed multiple independent simulations. For the 0% coverage case, we performed four independent 6 ns replicas, each using a different ML potential trained on the same data set. For finite coverage, 32 independent 6 ns simulations were carried out, sampling eight randomly generated adsorbate configurations, each repeated four times with different models. In this way, we can account for both the variance of the ML model and the statistical uncertainty associated with sampling. Because nitrogen diffusion is an activated process, extensive sampling is required to obtain statistically meaningful results. To ensure that the computed free energy profiles are not biased by specific initial conditions, and particularly the placement of nitrogen atoms, we employed a larger number of independent replicas. This approach yielded an aggregated simulation time of approximately 200 ns per coverage level.

### Enhanced Sampling Simulations

Standard MD simulations are inadequate for sampling rare events such as nitrogen recombination and subsurface migration, due to the high free energy barriers involved. To overcome this limitation and reconstruct the free energy profiles of these reactions, we need to employ enhanced sampling^23^ methods. In particular, we employed the On-the-fly Probability Enhanced Sampling (OPES) method,^51,52^ which constructs an adaptive bias potential in terms of selected collective variables (CVs), enabling efficient exploration of the relevant phase space while allowing reconstruction of the free energy landscape. To study N diffusion on the FeCo(110) surface, we consider the transformation of in-plane positions along the following crystallographic directions:

1 $$\begin{array}{ll} v_{11̅1} & =\frac{1}{a\sqrt{3}\;\cos\alpha \;\sin\alpha }\left(x\;\sin\alpha +y\;\cos\alpha \right)\\ v_{11̅1̅} & =\frac{1}{a\sqrt{3}\;\cos\alpha \;\sin\alpha }\left(-x\;\sin\alpha +y\;\cos\alpha \right) \end{array}$$

where *x* and *y* are the position along the [0 0 1] and [1 1̅ 0] directions, α is the angle between [1 1̅ 1] and [1 1̅ 0] directions, and *a* is the lattice parameter. Simulations of N diffusion on the surface used these CVs with an OPES barrier parameter of 45 kJ mol^–1^ and update interval of 500 steps. Harmonic walls (1000 kJ mol^–1^) were applied when |*v*| ≥ 1.6 to converge the free energy profile in a defined metastable region. To study the diffusion of N and H in the presence of another N*, additional reflective walls were applied at |*v*_*hkl*_| < 2.55 and |*v*_*hkl*_| < 0.55 to constrain the motion of the central atom.

For nitrogen recombination, the CV was chosen as the N–N distance *d*(N,N), starting from a precursor state with N atoms adsorbed at adjacent Fe and Co sites. To focus the sampling from the precursor state while allowing the N atoms to move across the surface, a symmetry-based wall was added, centered on the N–N center of mass:

2 $$v_{\mathrm{hkl}}^{\mathrm{abs}}=v_{\mathrm{hkl}}\left(∥d_{x}∥,∥d_{y}∥\right),\mathrm{hkl}=11̅1,11̅1̅$$

where *v*_*hkl*_ is defined by eq 1, and *d*_*i*_ represents the *x* and *y* Cartesian components of the distance between the two nitrogen atoms *d*(N,N). The harmonic constraint were applied when $v_{11̅1}^{\mathrm{abs}}>1.55$ and $∥v_{11̅1̅}^{\mathrm{abs}}∥>0.55$ . The OPES barrier parameter was set to 195 kJ mol^–^1 with a pace of 100 steps. The same parameters were adopted to perform the simulation at finite monolayer coverage except for the barrier parameter that was decreased to 190 kJ mol^–1^ and 185 kJ mol^–1^ for the coverage of 20% and 40%, respectively.

To study nitrogen migration into the subsurface, we use as CV the projection of the *N* position along the [0 1̅ 0] direction

3 $${ṽ}_{01̅0}=\frac{\sqrt{2}}{a}\left(-y+z\right)$$

This was used as CV to enhance the N migration, with an OPES barrier parameter of 220 kJ mol^–1^ (reduced to 210 kJ mol^–1^ and 200 kJ mol^–1^ at 20% and 40% coverages, respectively). To facilitate the readability of the results, in Figure 3 we report a transformation of the parameter *v*_01̅0_(*x*,*y*,*z*) = *ṽ*_01̅0_(*x*_*h*_,*y*_*h*_,*z*_*top*_) - *ṽ*_01̅0_(*x*,*y*,*z*), where *x*_*h*_,*y*_*h*_ = 0,0 is the planar position of the top hollow site occupied by nitrogen before dissolution and *z*_*top*_ is the average *z* coordinate of the FeCo top layer at 700 K. Harmonic restraints (with the same elastic constant as for surface diffusion) were applied to confine sampling to a single migration channel when ∥*ṽ*_11̅1_∥ > 0.55 and ∥*ṽ*_11̅1̅_∥ > 0.55, where the *ṽ* projections are defined as

4 $$\begin{array}{ll} {ṽ}_{11̅1} & =\frac{1}{a}\left(x+\frac{1}{\sqrt{2}}y+\frac{1}{\sqrt{2}}z\right)\\ {ṽ}_{11̅1̅} & =\frac{1}{a}\left(-x+\frac{1}{\sqrt{2}}y+\frac{1}{\sqrt{2}}z\right) \end{array}$$

For postprocessing, the coordination number between nitrogen and the metal atoms *C*_N,Fe|Co_ used in the analysis was defined using the same parameters of ref. 34.

### DFT Calculations

All DFT calculations for constructing the reference data set were performed using the PWscf code from the *Quantum ESPRESSO* suite.^53–55^ Exchange-correlation interactions were described using the PBE functional^56^ within the generalized-gradient approximation (GGA). Ultrasoft pseudopotentials from the SSSP PBE Precision v1.3.0 library^57^ were employed, treating 1, 5, 16, and 17 valence electrons for H, N, Fe, and Co, respectively.

The kinetic energy cutoff for the wave functions was set to 90 Ry, and 1080 Ry for the charge density. Collinear spin-polarized calculations were performed using the Marzari-Vanderbilt cold smearing scheme^58^ with a Gaussian width of 0.04 Ry. Initial spin polarizations were set to 0.6 for both Fe and Co atoms. Nonferromagnetic configurations with anomalously high energies were removed from the data set. Brillouin zone sampling was performed using a Monkhorst–Pack *k*-point grid^59^ with a maximum *k*-point spacing of 0.25 Å^–1^. Self-consistent field (SCF) calculations were considered converged when the total energy change was less than 10^–6^ Ry.

## Experimental Details

### Drying and Reduction of the Catalysts

For all measurements, 100 mg of catalyst sieve fraction (200–300 μm) was placed in a U-tube reactor fixed by two plugs of quartz wool. The spinel precatalysts MgFe_2_O_4_ and MgFeCoO_4_ were dried in a flow of He (80 mL_n_ min^–1^, 99.9999%) by heating from room temperature to 473 K with a rate of 5 K min^–1^ and holding this temperature for 1 h before cooling to 313 K. After drying, the catalyst was reduced in a flow of 7% H_2_/He (80 mL_n_ min^–1^, both 99.999%). The temperature program for the reduction consisted of heating to 873 K with a rate of 6 K min^–1^ and holding this temperature for 5 h before switching to He (50 mL_n_ min^–1^, 99.9999%) and cooling to 423 K.

### Transient NH_3_ Decomposition

During transient NH_3_ decomposition, the reduced catalysts were heated in a flow of 1.33% NH_3_/He (100 mL_n_ min^–1^, both 99.999%) to 873 K with a heating rate of 1 K min^–1^. The maximum temperature was held for 1 h before cooling to 423 K at the same rate as before. This transient NH_3_ decomposition cycle was repeated a second time for each sample.

### Desorption after NH_3_ Decomposition

To investigate the spent catalyst samples after NH_3_ decomposition, TPD profiles in He were measured. The sample was saturated with 1.33% NH_3_/He (100 mL_n_ min^–1^, both 99.999%) for 30 min at 423 K. Then, the flow was switched to 50 mL_n_ min^–1^ He (99.9999%) and the reactor was flushed for 30 min to remove NH_3_ from the gas phase. After flushing, the flow of He was reduced to 30 mL_n_ min^–1^ and the TPD experiment was performed with a heating rate of 5 K min^–1^ up to a temperature of 873 K.

### Kinetics Measurements

The kinetic measurements of ammonia decomposition were conducted in a BELCAT II catalyst analyzer (Microtrac Retsch GmbH). Prior to the catalytic steady-state measurements, 20 mg of the spinel (MgFe_2_O_4_ or Mg­(Fe_0.5_Co_0.5_)_2_O_4_) precatalyst (200–300 μm) were isothermally reduced for 5 h at 600 °C (7% H_2_/Ar, 80 mL_n_ min^–1^, β = 6 °C min^–1^, Air Liquide, H_2_ ≥ 99.999%, Ar ≥ 99.999%).

Afterward, the freshly reduced samples were cooled down to 500 °C and exposed for 5 h to a mixture of 3% NH_3_ in Ar (40 mL_n_ min^–1^, Air Liquide, NH_3_ ≥ 99.999%, Ar ≥ 99.999%) to allow nitridation. Then, steady-state catalytic ammonia decomposition measurements were conducted between 400 and 600 °C. First, the samples were cooled down to 400 °C at a gas flow of 80 mL_n_ min^–1^ (3% NH_3_/Ar), which was directed through the catalyst bed. After measuring at 400 °C, the samples were heated up stepwise to 425, 450, 475, 500, 550, and 600 °C and held at each temperature for 3 h.

A Micro GC Fusion from Inficon was calibrated for NH_3_, N_2_, and H_2_ to perform analysis of the exhaust gas. The mole and corresponding volume changes of the gas mixture due to reaction and its effect on the conversion data were neglected, as it is limited to only 3% NH_3_ in a 97% Ar diluent fraction. The metal-mass-normalized H_2_ production rates and the apparent activation energy (*E*_a_) were calculated at low NH_3_ conversion, approaching differential reaction conditions.

### Long-Term Stability Measurements

The setup for long-term measurements consisted of a gas supply for pure N_2_, H_2_, Ar, and NH_3_. A 1/2” stainless steel reactor, coated with Silcolloy by SilcoTek, was used. Four type K thermocouples, each with a diameter of 0.25 mm, were inserted at 2 cm intervals into a 1/8” stainless steel tube, which served as a protective housing and were placed into the reactor. This configuration allows for measuring the temperature along the entire catalyst bed. A three-zone furnace (HTM Reetz) with a maximum operating temperature of 1000 °C was used for temperature control. All gas lines were heated to 150 °C to prevent condensation. Gas analysis consisted of a Agilent of a 8860 gas chromatograph and a multichannel gas analyzer (X-Stream XEPG from Emmerson).

To perform the experiment, 0.5 g of the catalyst was diluted with 4.5 g of SiC, both sieved to a particle size of 250–355 μm. The total gas flow for all steps was set to 500 mL min^–1^. Prior to the measurements, the catalyst was pretreated at 200 °C for 2 h in N_2_. For the reduction step, a gas mixture of 6% H_2_/N_2_ was used and the catalyst was heated from 100 °C to 600 °C at a rate of 5 K min^–1^ followed by a 5 h holding period at 600 °C. After reduction, the catalyst was cooled to 150 °C and 98% NH_3_/Ar was introduced. A single temperature-programmed cycle for NH_3_ decomposition was performed from 150 °C to 750 °C at a heating rate of 1 K min^–1^. The long-term measurement was performed at 90% NH_3_ conversion for a total duration of 1000 h. Therefore, the furnace temperature was set to 628 °C, corresponding to 558 °C as measured by the thermocouple inside the catalyst bed. NH_3_ mole fractions were obtained by GC analysis. The NH_3_ conversion *X* was calculated using

5 $$X=\frac{1-\frac{y_{NH_{3}}}{y_{NH_{3}}^{0}}}{1+y_{NH_{3}}}$$

where *y*_NH_3__ is the NH_3_ mole fraction obtained by GC analysis. The calculation considers the volume expansion during the reaction using high initial NH_3_ mole fractions $y_{NH_{3}}^{0}$ at high conversion.

## Supplementary Material



## Data Availability

The outputs of the molecular dynamics simulations reported in this paper are available from the corresponding author (L.B.) upon request.

## Notes

A: Supporting information and validation on ML-based interatomic potential. B: Additional computational results. C: Materials and catalysts characterization. D: Additional experimental results (PDF)
