# Supplementary material for: No Time for Nitrides: How Cobalt Alloying Promotes Iron Catalysts for Ammonia Decomposition
Source: ACS Catal. 2025 Sep 17;15(19):16690–702. doi: 10.1021/acscatal.5c04795 (PMC12622394; doi:10.1021/acscatal.5c04795)
Supplement: Supplementary file 1 [file cs5c04795_si_001.pdf]

Supporting Information

No Time for Nitrides: How Cobalt Alloying Promotes Iron Catalysts for Ammonia Decomposition

Simone Perego,[1] Maximilian Purcel,[2,3] Yannick Baum,[4] Shilong Chen,[4,5,*)] Astrid Sophie Müller,[2] Michele Parrinello,[1] Malte Behrens,[4,6] Martin Muhler,[2,3] and Luigi Bonati[1,*)]
[1)] *Atomistic Simulations, Italian Institute of Technology, 16163 Genova, Italy*
[2)] *Laboratory of Industrial Chemistry, Ruhr University Bochum, 44780 Bochum, Germany*
[3)] *Max Planck Institute for Chemical Energy Conversion, 45470 Mülheim an der Ruhr, Germany*
[4)] *Institute of Inorganic Chemistry, Kiel University, 24118 Kiel, Germany*
[5)] *National Engineering Research Center of Chemical Fertilizer Catalyst (NERC-CFC), School of Chemical Engineering, Fuzhou University, 350002 Fuzhou, China*
[6)] *Kiel Nano, Surface and Interface Science KiNSIS, Kiel University, 24118 Kiel, Germany*

Appendix A: ML-based interatomic potential

1. Data-Efficient Active Learning (DEAL) of the Machine Learning Potential

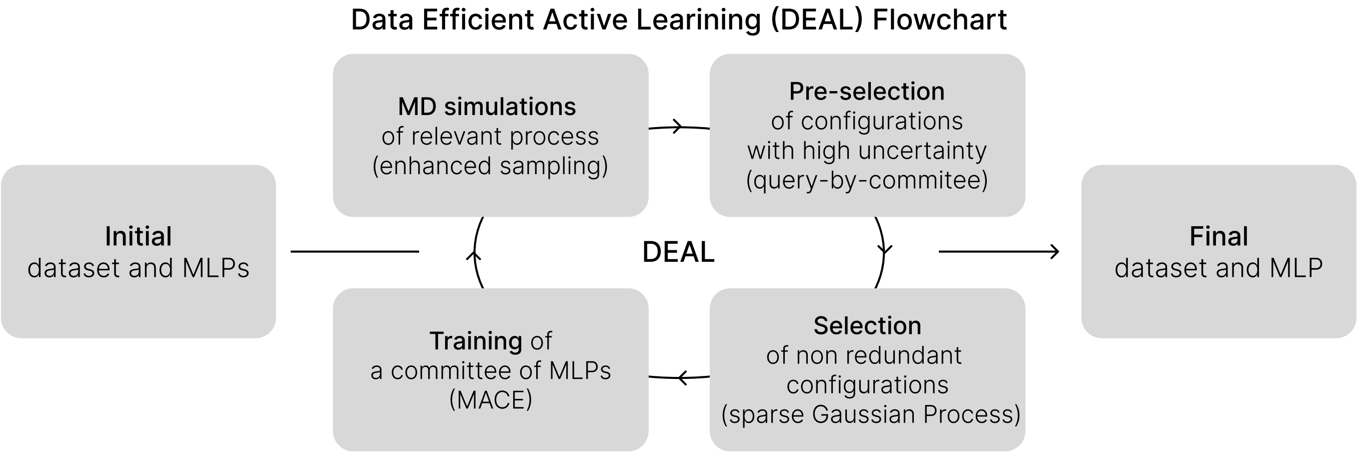

Figure S1. Flowchart of Data-Efficient Active Learning (DEAL) protocol used for constructing reliable reactive Machine learning potential. The procedure, originally developed in Ref. 1, is described in detail in the Method section.

To capture lateral interactions under operando conditions, we performed five active learning iterations using the DEAL framework. We adopted the variant where the Gaussian Process (GP) model is retrained from scratch at each cycle, and DFT calculations are distributed in an embarrassingly parallel manner, maximizing computational efficiency[1].

Although lateral interactions introduce considerable complexity, the initial ML potential already included configurations of isolated adsorbates, enabling DEAL to start from a reasonably accurate baseline.

In the first iteration, the base model (MLP0) was used to run short unbiased MD simulations (100 ps) across varying adsorbate coverages (17%, 33%, 50%) and compositions (N, H, N/H = 1:1 and 1:3). Since all sampled configurations were new to the model, we opted for a uniform sampling strategy, pre-selecting one frame every 2.5 ps (without using the query-by-committee criterion).

---

*) Email: schen@fzu.edu.cn, luigi.bonati@iit.it

Subsequent iterations followed the standard DEAL protocol: (a) pre-selection with query-by-committee, (b) screening via GP-based diversity selection, (c) DFT single-point calculations of the selected structures, (d) retraining of the MLP with the updated dataset.

After two initial non-reactive iterations, we targeted the recombination and sub-surface migration reactions using longer reactive MD trajectories ($\geq$1 ns) at coverages of 33% and 50% (N:H = 1:1 and 1:3). These yielded the final model (MLP5).

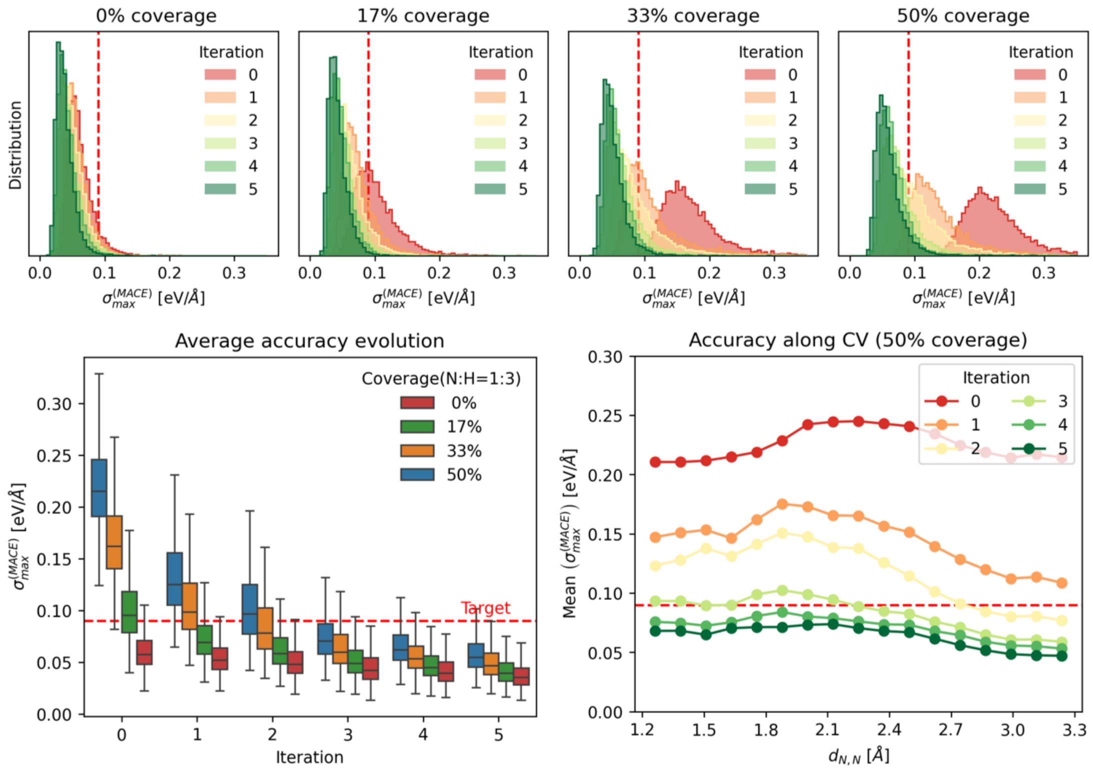

Figure S2. Evolution of MLP accuracy during active learning iterations for N–N recombination reactions at various coverages (17%, 33%, and 50% monolayer, with N:H = 1:3) at 700 K. Top: distributions of the maximum force uncertainty $\sigma_{\max}^{\mathrm{MACE}}$ along sampled reactive trajectories. Bottom left: boxplots of $\sigma_{\max}^{\mathrm{MACE}}$ per iteration. Bottom right: average uncertainty along the reaction coordinate ($d_{\mathrm{N,N}}$) for 50% coverage.

Figures S2 and S3 track the evolution of model accuracy across iterations and reaction pathways. Notably, in high coverage scenarios, more learning cycles were needed to reach the 90 meV/Å uncertainty threshold, reflecting the increased configurational complexity. The bottom right panels show that initial iterations improve the description near reactants and products, but transition states (TS) remain poorly captured until later cycles. Only after targeted sampling of reactive events the uncertainty drops uniformly along the reaction path.

Selected configurations during DEAL iterations

Iteration	1	2	3	4	5	**Total**
Pre-selected	3863	2606	8089	24297	11086	49941
GP-selected	194	252	911	755	917	**3029**

Table S1. DEAL configurations selection during the five iterations targeting finite coverage effects. The GP threshold was set to 0.1, except for iteration 4 (0.12) due to a large preselection pool.

In total, 3029 DFT configurations were collected and added to the training set during these five iterations (Table S1). Including earlier data, the final ML potential was trained on approximately 8200 configurations, highlighting the data efficiency of this approach.

Beyond accuracy, the DEAL workflow also proved practical. Each cycle is completed within 1–2 days using modest resources. The MD simulations ran efficiently on a single GPU, while GP selection and retraining were performed in under 24 hours using a few dozen CPUs. DFT recalculations were fully parallelized, keeping wall time minimal.

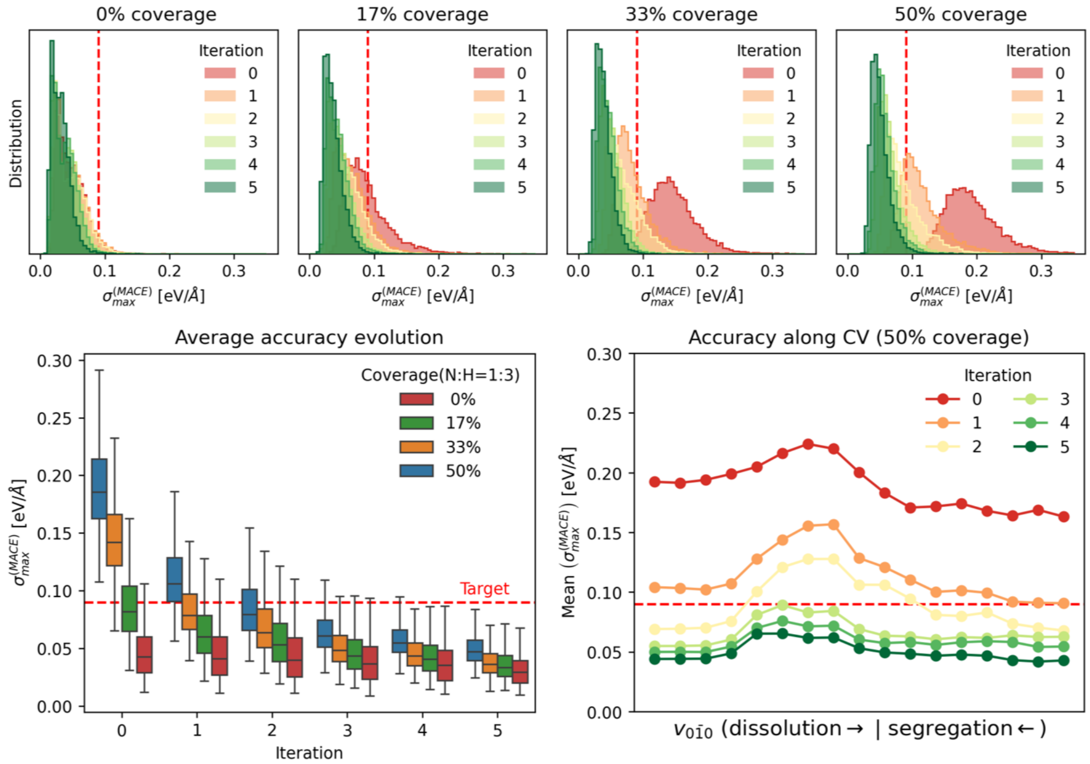

Figure S3. Analogous to Figure S2 for the nitrogen dissolution/segregation reaction.

2. Dataset composition

The protocol used for the construction of the (original) dataset employed to study the decomposition of NH_3 on FeCo(110) is described in detail in Ref. 1. In this work, the dataset was expanded to study nitrogen recombination and dissolution/segregation in the presence of lateral interactions using DEAL as described in Methods. The resulting final dataset comprises about 8200 configurations. The detailed composition is reported in Tab. S2.

FeCo original dataset composition[1]

Formula	Adsorbate	N. config.	Formula	Adsorbate	N. config.
$Co_{30}Fe_{30}H_2$	H	251	$Co_{60}Fe_{60}HN$	NH	618
$Co_{30}Fe_{30}H_2N_2$	NH	301	$Co_{60}Fe_{60}N$	N	10
$Co_{30}Fe_{30}H_4N_2$	NH_2	173	$Co_{60}Fe_{60}N_2$	2 N	84
$Co_{30}Fe_{30}H_6N_2$	NH_3	125	$Co_{60}Fe_{60}N_2$	N	17
$Co_{30}Fe_{30}N_2$	N	275	$Co_{60}Fe_{60}N_2$	N_2	86
$Co_{30}Fe_{30}N_4$	2 N	157	$Co_{60}Fe_{60}N_4$	2 N	53
$Co_{30}Fe_{30}N_4$	N_2	140	$Co_{60}Fe_{60}N_4$	N_2	9
$Co_{60}Fe_{60}H_2$	H	13	$Co_{72}Fe_{72}$	–	130
$Co_{60}Fe_{60}H_2N$	NH_2	1236	$Co_{72}Fe_{72}H_4N_2$	NH_2	130
$Co_{60}Fe_{60}H_2N_2$	NH	25	$Co_{72}Fe_{72}H_6N_2$	NH_3	129
$Co_{60}Fe_{60}H_3N$	NH_3	886	$Co_{72}Fe_{72}N$	N	181
$Co_{60}Fe_{60}H_4N_2$	NH_2	8	$Co_{72}Fe_{72}N_2$	2 N	130
$Co_{60}Fe_{60}H_6N_2$	NH_3	29			
**Total**					**5193**

Dataset expansion for lateral interactions

Formula	N. config.	Formula	N. config.
$Co_{60}Fe_{60}H_{12}N$	29	$Co_{60}Fe_{60}H_8N$	28
$Co_{60}Fe_{60}H_{12}N_2$	34	$Co_{60}Fe_{60}H_8N_2$	14
$Co_{60}Fe_{60}H_2N_3$	3	$Co_{60}Fe_{60}H_9N_4$	45
$Co_{60}Fe_{60}H_2N_4$	15	$Co_{60}Fe_{60}H_9N_5$	568
$Co_{60}Fe_{60}H_3N_2$	2	$Co_{60}Fe_{60}N_{10}$	7
$Co_{60}Fe_{60}H_3N_3$	26	$Co_{60}Fe_{60}N_{13}$	7
$Co_{60}Fe_{60}H_3N_5$	54	$Co_{60}Fe_{60}N_6$	3
$Co_{60}Fe_{60}H_4N$	3	$Co_{60}Fe_{60}N_9$	8
$Co_{60}Fe_{60}H_4N_2$	4	$Co_{72}Fe_{72}H_2N_3$	3
$Co_{60}Fe_{60}H_4N_5$	9	$Co_{72}Fe_{72}H_3N_2$	3
$Co_{60}Fe_{60}H_4N_6$	170	$Co_{72}Fe_{72}H_3N_4$	25
$Co_{60}Fe_{60}H_5N_7$	301	$Co_{72}Fe_{72}H_4N_5$	82
$Co_{60}Fe_{60}H_6N_3$	14	$Co_{72}Fe_{72}H_5N_6$	277
$Co_{60}Fe_{60}H_6N_4$	103	$Co_{72}Fe_{72}H_6N_3$	44
$Co_{60}Fe_{60}H_6N_7$	83	$Co_{72}Fe_{72}H_6N_7$	234
$Co_{60}Fe_{60}H_6N_8$	309	$Co_{72}Fe_{72}H_7N_4$	47
$Co_{60}Fe_{60}H_7N_5$	99	$Co_{72}Fe_{72}H_9N_4$	376
**Total**			**3029**

Table S2. Composition of the configuration in the dataset for training and validation of the potential divided by chemical formula and adsorbate type. The first block refers to the original data collected in Ref. 1. The second part refers to the database expansion conducted in this work.

3. Validation of Machine Learning Potential

To demonstrate the reliability of MLP in performing DFT-quality simulation, we conducted two types of validation. The first one, reported in Fig. 1, is conducted on an independent dataset composed of configurations with different concentrations of adsorbates (ranging from 0 to 50% monolayer coverage, N:H=1:3). As mentioned before, the final MLP is able to achieve uniform accuracy on the description of all the different environments, especially reactive ones, with a mean absolute error of 0.31 meV/atom in energy and 12 meV/Å in force predictions. Secondly, to evaluate the

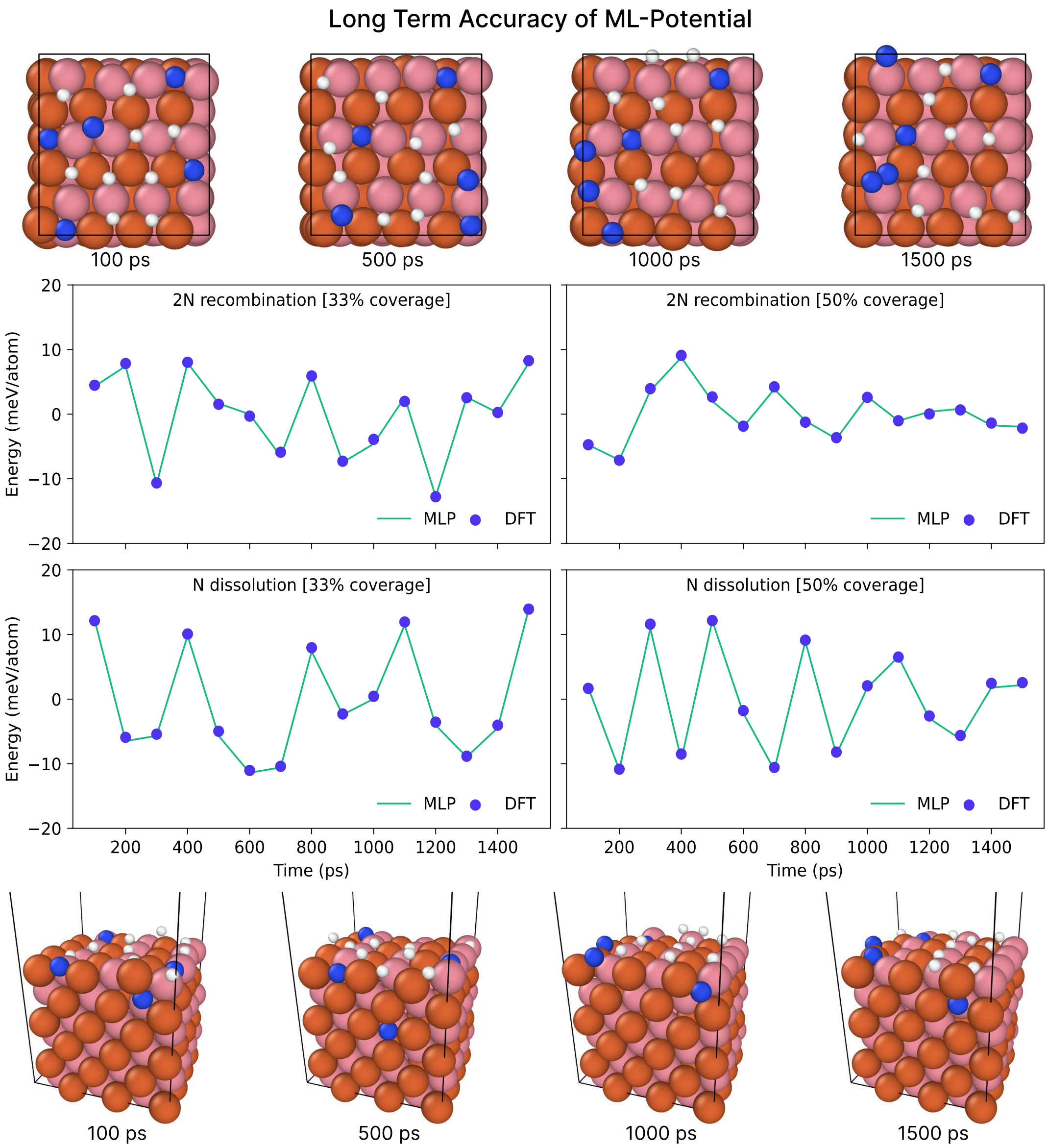

Figure S4. Comparison of energy prediction of the MLP and DFT for selected snapshots extracted from 1.5 ns-long reactive simulations of the N-N recombination (top panels) and N dissolution (bottom panels). The simulations are performed under high coverage conditions (33% and 50% monolayer coverage, N:H=1:3), as these represent the most critical simulation environments. Some snapshots of the configurations selected are also reported, over the top panels for N-N recombination and below the bottom panels for N dissolution (both at maximum coverage). The quality of the MLP prediction is maintained along all the simulations, with an overall mean absolute error of 0.30 meV/atom in energy and 13 meV/Å in force predictions.

accuracy and stability of the potential during MD simulations, especially when large external biasing forces are applied, we perform 1.5 ns of reactive simulations (recombination and dissolution) on a small FeCo substrate (containing 120 and 144 FeCo atoms, respectively), and re-evaluate the configuration at DFT level every 100 ps. The simulations are performed under high coverage conditions (33% and 50% monolayer coverage, N:H=1:3), as these represent the most critical simulation environments. As shown in Figure S4, the accuracy of the MLP is maintained along all the simulations, with an overall mean absolute error of 0.30 meV/atom in energy and 13 meV/Å in force predictions on the recalculated configuration.

Appendix B: Additional Computational Results

1. Nitrogen diffusion

Here, we report the study of nitrogen diffusion on the FeCo(110) surface that we conducted prior to the study of 2 N recombination and N dissolution/segregation. In the top left panel of Figure S5, we present the 2D free energy surface (FES) projected along the two in-plane crystallographic directions [0 0 1] (x) and [1 $\bar{1}$ 0] (y). We can immediately observe that nitrogen is preferentially adsorbed at the hollow sites (h). However, the FeCo(1 1 0) surface features two distinct hollow sites, h_{Fe} and h_{Co}, due to the asymmetry caused by the two different chemical species. In the h_{Fe} site, nitrogen is coordinated with two cobalt atoms (in the first layer along the x direction) and three iron atoms (two in the first layer along the y direction and one in the layer below). Similarly, in the h_{Co} site, the coordination is the same but with iron and cobalt swapped. From the minimum free energy pathway, we see that h_{Fe} is slightly favored over h_{Co}, which has a free energy that is approximately $2\,k_B$T higher.

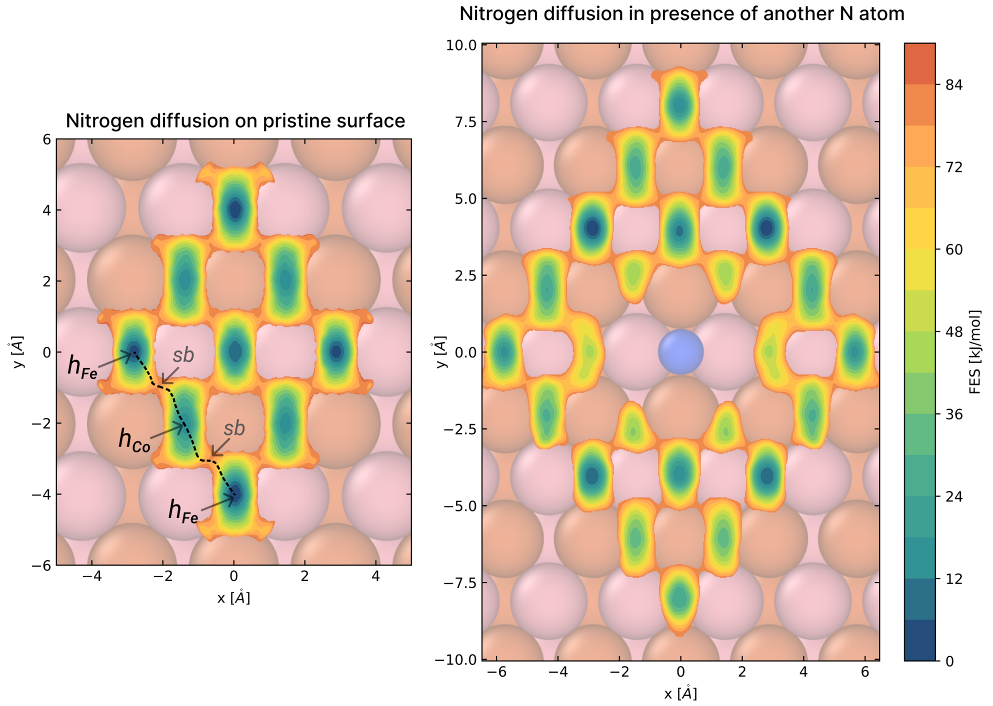

Figure S5. On the left panel, free energy of N^* projected along the two crystallographic directions [0 0 1] (x-axis) and [1$\bar{1}$0] (y-axis). Local minima represent metastable states, and black dotted lines denote the minimum free energy diffusion pathways. The high-symmetry adsorption sites are labeled according to Ref. 2. On the right, the same free energy surface, in the presence of another atomic nitrogen adsorbed in the center. For an effective sampling, a reflective wall was placed at $|v_{hkl}| < 2.55$, $hlk = 1\bar{1}1, 1\bar{1}\bar{1}$, at 1.55 in the absence of the second N^* atom. Similarly, to avoid the diffusion of the central nitrogen, another wall at $|v_{hkl}| < 0.55$, $hlk = 1\bar{1}1, 1\bar{1}\bar{1}$.

In addition, the FES reveals the diffusion mechanism and the associated free energy barrier. The minimum free energy path (black dotted line) for diffusion between adjacent sites passes through a short bridge (sb); in this case, all sb sites are equivalent. Specifically, to move from one h_{Fe} site to the next, nitrogen passes through an h_{Co} site, crossing two sb sites:

$$h_{\mathrm{Fe}} \xrightarrow{sb} h_{\mathrm{Co}} \xrightarrow{sb} h_{\mathrm{Fe}},$$

with free energy barriers ($\Delta G^{\ddagger}$) of approximately 75 and 65 kJ/mol, respectively (Figure S5, left panel).

Furthermore, the study of the nitrogen diffusion in the presence of another adsorbed N* at the center (Figure S5, right panel). As can be observed, the four hollow sites adjacent to the occupied central site are approximately 40 kJ/mol higher in energy than the most stable sites. Once again, these sites are of considerable interest because they represent the precursor states of recombination. It should be noted that in the specific case depicted in the figure, the central nitrogen is placed in h_{Fe}, and the four adjacent sites are h_{Co}. However, the result remains the same if the central atom is placed in h_{Co}, as the presence of two nitrogen atoms in two adjacent h sites resolves the asymmetry of the problem.

2. Nitrogen interaction with the substrate

Table S3 reports the total charge transferred to a nitrogen atom when adsorbed either in hollow sites or in octahedral interstitial sites (at a depth of three layers, $v_{0\bar{1}0} \approx 4$). As expected, the net charge transferred to N is larger when the atom is located in Fe-rich environments, consistent with the higher electronegativity of Co. For comparison, values for the pure Fe(110) surface are also reported. In this case, the net charge transferred to N is consistently higher than in the alloy.

All results were obtained at the DFT level (see Methods for details), after geometry optimization using the BFGS quasi-Newton algorithm with a force convergence criterion of $0.0257\,\mathrm{eV\,Å^{-1}}$, to eliminate the noise due to high-temperature thermal fluctuation.

Charge transferred to N atom [e]

	Hollow		Octahedral	
	h_{Fe}	h_{Co}	O_{Fe}	O_{Co}
FeCo	-1.120	-1.107	-1.200	-1.179
Fe	-1.155		-1.236	

Table S3. Charge transferred to a nitrogen atom adsorbed in different sites on FeCo and pure Fe catalysts. Results are from DFT calculations (see Methods), after geometry optimization.

3. Nitrogen recombination

In Fig. S6, we report the the analogous of Fig. 2, but here we projected the FES on the distance between two nitrogen atoms $d(\mathrm{N,N})$ and the coordination between nitrogen and the metal atoms $C_{\mathrm{N,\,Fe|Co}}$.

The reactant state consists of two N^* atoms adsorbed at two adjacent h sites, one at h_{Fe} and one at h_{Co}, while the transition state (TS) is located near the *sb* site (see snapshots on the right). For this reason, we do not report results resolved for the coordination numbers with Fe and Co, as they are identical by symmetry. Instead, in the product states, the adsorption states for N_2, horizontal (α) and vertical (γ), are degenerate and could be resolved based on the two different metals. However, they are similar in energy and are of little interest to the recombination reaction, as once $N_2{}^*$ is formed, it can easily desorb from the surface.

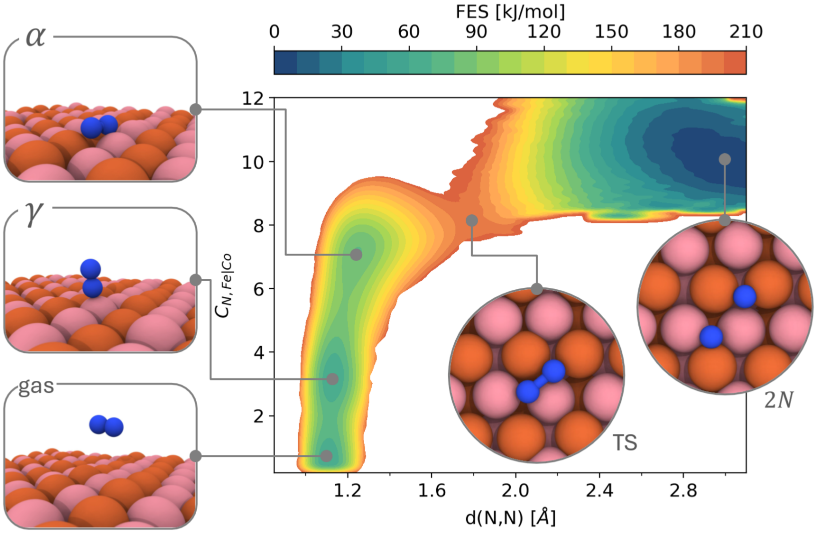

Figure S6. Free energy surface (FES) of nitrogen recombination/dissociation ($2\,\mathrm{N}^* \rightleftharpoons \mathrm{N_2}$) as a function of N-N distance $d(\mathrm{N,N})$ and the coordination between nitrogen and the metal atoms $C_{\mathrm{N,\,Fe|Co}}$ at 700 K. The local minima on the plots correspond to metastable states. Some snapshots along the reaction are reported. From right to left: reactants (2 N), transition state (TS), various N_2 adsorption sites (α, and γ) and products (N_2 gas phase).

4. Effect of lateral interactions on free energy barriers

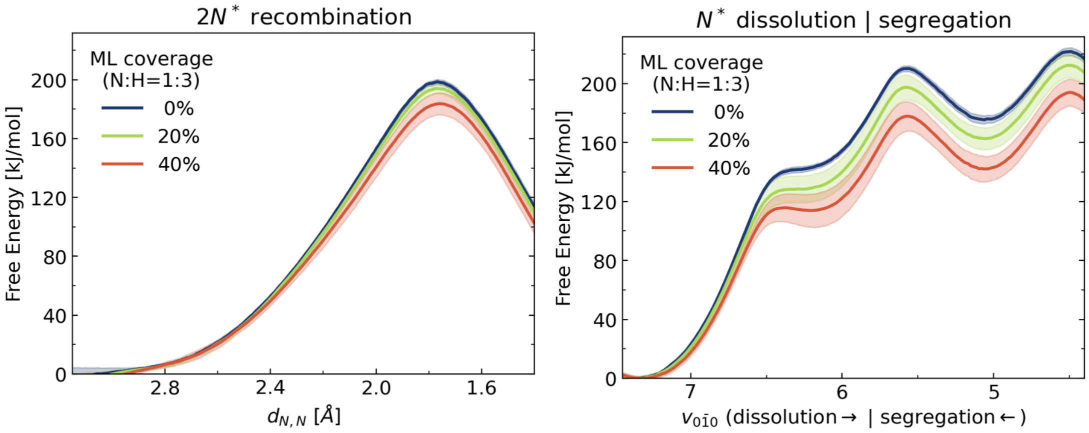

Figure S7. Free energy profiles of nitrogen recombination (left) and dissolution/segregation (right) on the FeCo(110) slab at T=700 K at various coverages (0, 20, and 40% of a monolayer with an N : H = 1 : 3).

Appendix C: Materials and characterization

1. Starting Materials

For the synthesis of the catalyst precursors (layered double hydroxides, LDHs) of the spinel samples, the following commercially available chemicals were used without further purification: $Co(NO_3)_2 \cdot 6\,H_2O$ (99%, Grüssing GmbH), $FeSO_4 \cdot 7\,H_2O$ (99%, Grüssing GmbH), $Fe(NO_3)_3 \cdot 9\,H_2O$ (99%, Grüssing GmbH), $Mg(NO_3)_2 \cdot 6\,H_2O$ (99%, Grüssing GmbH), Na_2CO_3 (99.5%, Grüssing GmbH), and NaOH (99%, Grüssing GmbH).

2. Synthesis

Spinel $Mg(Fe_{1-x}Co_x)_2O_4$ ($x = 0$ and 0.5) pre-catalysts were synthesised through co-precipitation of LDHs in an automated laboratory reactor system (Optimax synthesis workstation, Mettler Toledo). During co-precipitation, the metal solution contained three equal concentrations of Mg^{2+}, Fe^{3+}, and M^{2+} (M = Fe or Co) of 0.266 mol L^{-1}, yielding $Mg(Fe_{1-x}Co_x)_2O_4$ with either x = 0 or 0.5. A mixed aqueous 0.6 mol L^{-1} NaOH and 0.09 mol L^{-1} Na_2CO_3 solution served as the precipitation agent. The pH during co-precipitation was kept constant at 10.0 for $Mg(Fe_{0.5}Co_{0.5})_2O_4$ with an aging time of 1 h at 50 °C, and 10.5 for $MgFe_2O_4$ with an aging time of 24 h at 50 °C. After washing with water until the conductivity of the supernatant was below 100 µS cm^{-1}, and drying, the precursors were calcined at 600 °C for 3 h and further isothermally reduced in H_2 prior to the reaction.

3. Characterization

Iron, cobalt, and magnesium contents in the samples were determined by Inductively Coupled Plasma Optical Emission Spectroscopy (ICP-OES) (Avio 200, PerkinElmer).

N_2 adsorption-desorption experiments of the LDH and spinel precursors were conducted with a NOVA3000e setup (Quantachrome Instruments) at -196 °C after degassing the samples at 80 °C for 3 h in vacuum. BET (Brunauer–Emmett–Teller) surface areas were calculated from p/p_0 data between 0.05 and 0.3. Pore volumes and average pore sizes were determined by applying the BJH (Barrett–Joyner–Halenda) method.

The results of these characterizations are reported in Tables S4 and S5.

Table S4. BET specific surface area, pore volume, and pore size of Mg-Fe-Co-based LDH/hydroxide and the spinel pre-catalysts.

**Catalyst**	**Surface area ($m^2\,g^{-1}$)**	**Pore volume ($cm^3\,g^{-1}$)**	**Average pore size (nm)**
$MgFe_2$-LDH	47.8	0.32	27.4
$MgFe_2O_4$	64.0	0.41	25.6
$Mg(Fe_{0.5}Co_{0.5})_2$-LDH	71.4	0.49	27.5
$Mg(Fe_{0.5}Co_{0.5})_2O_4$	54.8	0.65	46.7

Table S5. ICP results: The atomic ratios of metal elements of different catalysts. The nominal values are given in parentheses.

**Sample**	**Atomic ratio/(Fe+Co+Mg)%**		
	**Mg**	**Fe**	**Co**
**$MgFe_2O_4$**	32.6	67.4	0
**$Mg(Fe_{0.5}Co_{0.5})_2O_4$**	32.7	33.7	33.6

Powder X-ray diffraction (XRD) patterns of the samples were measured at room temperature with a STOE Stadi-P diffractometer, equipped with a MYTHEN 1K detector from Dectris using molybdenum $K\alpha_1$ radiation. The XRD samples for precursor and calcined materials were prepared between scotch tape and measured in transmission geometry. To avoid re-oxidation of the spent catalysts, sample preparation was performed in an Ar glovebox, where the catalysts were sealed into capillaries with a diameter of 0.7 mm and further measured in Debye–Scherrer geometry.

In Fig. S8 we report the XRD patterns for the co-precipitated precursor, the calcinated pre-catalyst (before reduction), and the spent catalyst (after reduction and reaction). If we compare the phase composition of the Fe/MgO sample used in this study with the previous batch reported in Ref. 3, we note small differences in phase compositions, although

the samples were synthesised using the same nominal procedure. Specifically, our sample contained a minor hematite phase in the pre-catalyst, which was absent in the earlier one. Moreover, although Fe_3N remains the dominant phase in the spent catalysts, it additionally exhibits the formation of Fe_4N. In contrast, the bimetallic FeCo/MgO catalyst reproduced under the same synthesis conditions showed excellent consistency in structure and activity compared to earlier results, underlining the robustness of this formulation. The higher sensitivity of Fe/MgO to batch-specific conditions likely arises from the involvement of aqueous Fe^{2+} chemistry during synthesis, which is more susceptible to oxidation and harder to control than the synthesis of the bimetallic system.

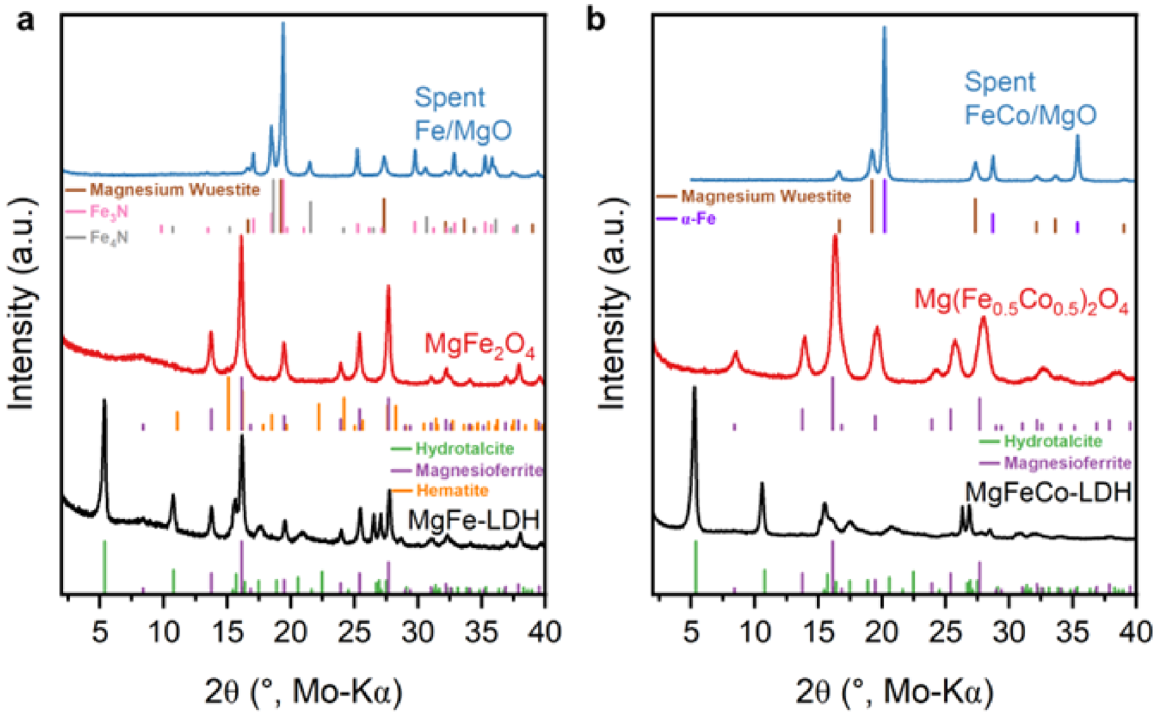

Figure S8. XRD patterns of LDH precursor, calcined pre-catalyst (before reduction), and the spent catalyst (after ammonia decomposition reaction) from bottom to top for Fe/MgO (a) and FeCo/MgO (b), respectively. References: Magnesioferrite (ICSD: 41290), Hydrotalcite (ICSD: 182294), Hematite (ICSD: 40142), α-Fe (ICSD: 52258), magnesium wuestite (ICSD: 181215), Fe_3N (ICSD: 20389), Fe_4N (ICSD: 60195).

Scanning electron microscope (SEM) studies of the spent FeCo/MgO catalysts were carried out with a Hitachi SU8700 microscope with inert gas-sample-trasfer function, equipped with an InLens-Detector, an Everhart-Thornley Detector, and a UVD-Dual-Detector for secondary electron detectors, together with a Photodioden detector for backscattering electron detectors. The Oxford EDX-Detektor Ultim Max 100 is employed for the Energy-Dispersive X-ray (EDX) analysis to produce elemental composition maps.

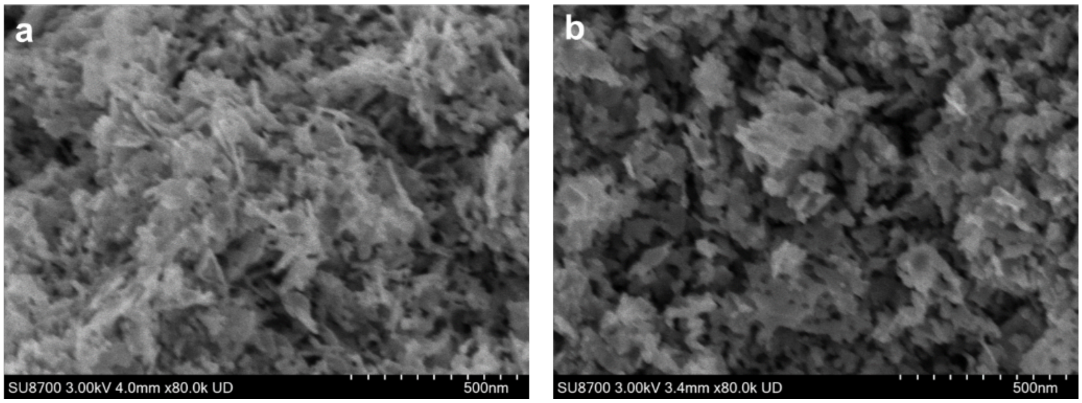

Figure S9. SEM images (a, b) of the FeCo/MgO catalyst after ammonia decomposition

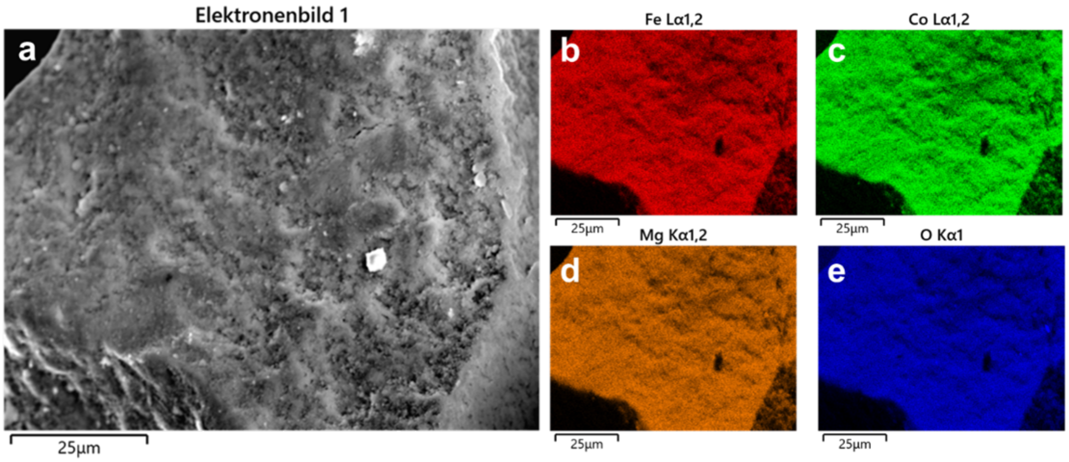

Figure S10. SEM image (a) and the corresponding EDX maps of the FeCo/MgO catalyst after ammonia decomposition, where the Fe & Co elements (b & c) are related to L-line intensities and the Mg & O elements (d & e) are related to K-line intensities.

Appendix D: Additional Experimental Results

1. Steady-state kinetic measurements

Fig. S11 reports the results of the steady-state NH_3 decomposition under differential reaction conditions with 3 % NH_3/Ar at a flow rate of 80 $ml_n\,min^{-1}$ for Fe/MgO and FeCo/MgO catalysit. The relative Arrhenius fit from which an apparent activation energy of ~150 $kJ\,mol^{-1}$ and ~104 $kJ\,mol^{-1}$, for Fe/MgO and FeCo/MgO respectively, is also reported in the figure.

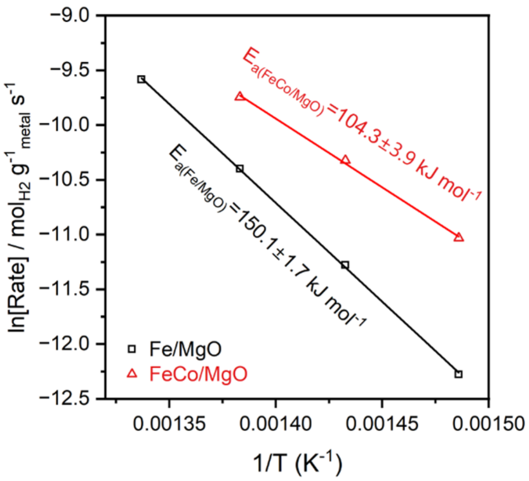

Figure S11. Arrhenius plots of Fe/MgO and $Fe_{0.5}Co_{0.5}$/MgO catalysts for ammonia decomposition reaction under differential reaction conditions with 3 % NH_3/Ar at a flow rate of 80 $ml_n\,min^{-1}$. The error associated with the apparent activation energy, reported in the figure, represents only the linear fitting analysis error.

Additionally in Fig. S12 we report the comparison of the NH_3 conversion during the steady-state NH_3 decomposition under 3 % NH_3 at a flow rate of 80 $ml_n\,min^{-1}$ for Fe/MgO catalysts used in the current work (blu circle) and a previous batch reported in Ref. 3 (red triangle). Fe/MgO-current catalyst exhibits a similar NH_3 conversion to that of Fe/MgO-previous catalyst in the overall range of the reaction temperature.

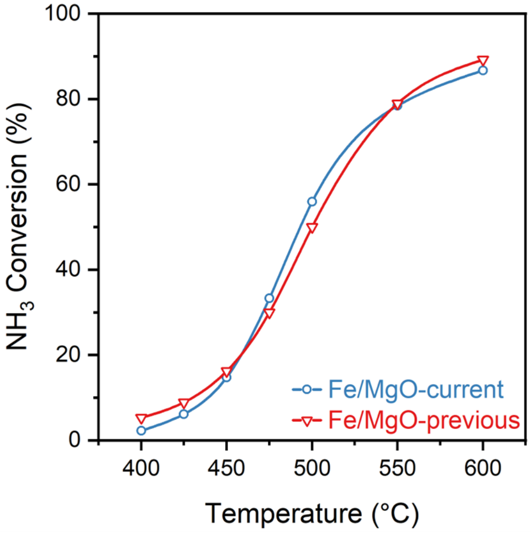

Figure S12. NH_3 conversion of the two batches of Fe/MgO catalysts in ammonia decomposition in a gas mixture with 3 % NH_3 at a flow rate of 80 $ml_n\,min^{-1}$. The Fe/MgO-previous catalyst derived from $MgFe_2O_4$ was synthesized at the University of Duisburg-Essen and reported previously[3], while the Fe/MgO-current catalyst derived from $MgFe_2O_4$ was synthesized at Kiel University and is reported in this study.

2. Desorption after NH_3 decomposition

In Fig. S13 we report the desorption profiles of H_2 and NH_3 after transient NH_3 decomposition and 30 min NH_3 exposure at 423 K for the Fe/MgO and FeCo/MgO. Here, we observe the absence of H_2 and NH_3 release on both catalysts.

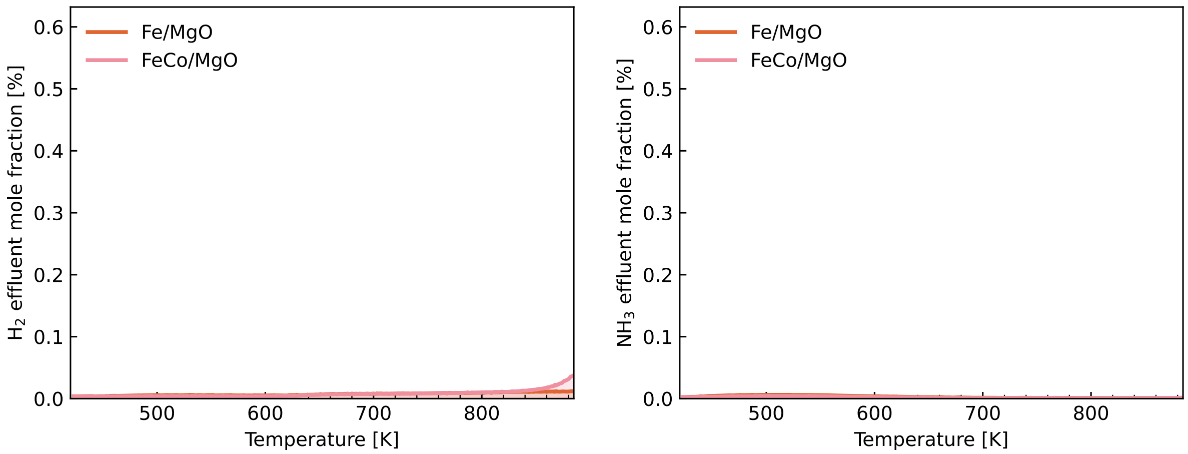

Figure S13. Desorption profiles of H_2 and NH_3 from Fe/MgO and FeCo/MgO subsequent to 30 min NH_3 exposure at 423 K (pure He, 423 K-873 K, $\beta = 5\,\mathrm{K\,min^{-1}}$).

3. Long-term measurements

In Fig. S14 we show the long-term performance of ammonia decomposition over the FeCo/MgO catalyst. The catalyst exhibits a high initial conversion of ~88% (at 558°C, measured inside the catalyst bed) and maintains stable activity over extended operation, with only a gradual decline of ~ 6% along the test time. Overall, the activity loss at 558 °C corresponds to just 0.67%/100 h relative to the maximum conversion. For comparison, under similar conditions (at 534 °C), reported values are 0.19%/100 h for a Ni-based reference catalyst and up to 1.69%/100 h for an unpromoted Ni/Al_2O_3 catalyst[4].

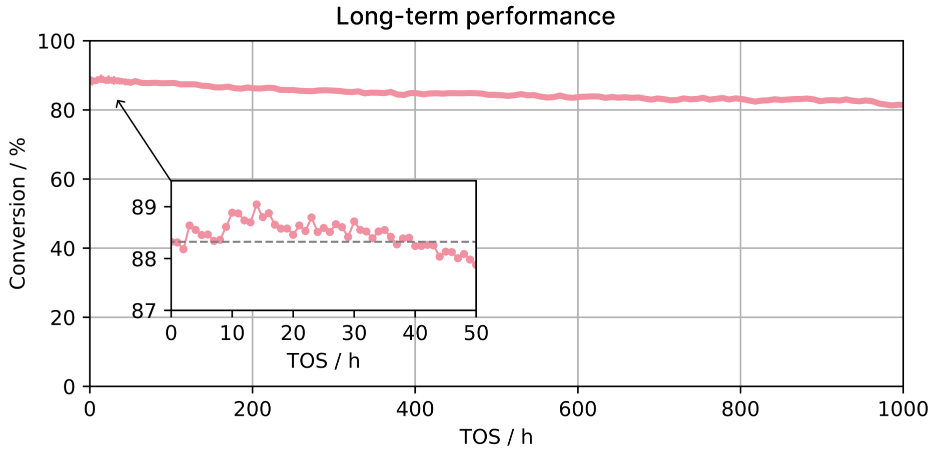

Figure S14. Ammonia decomposition over the FeCo/MgO catalyst during 1000 h time on stream (TOS) under a feed of 98% NH_3 in Ar at 558°C (measured inside the catalyst bed). The inset highlights the first 50 h of TOS, showing a brief activation phase with a ~1% increase in conversion.

REFERENCES

[1] S. Perego and L. Bonati, "Data efficient machine learning potentials for modeling catalytic reactivity via active learning and enhanced sampling," npj Computational Materials 2024 10:1 **10**, 1–13 (2024).

[2] S. Perego, L. Bonati, S. Tripathi, and M. Parrinello, "How Dynamics Changes Ammonia Cracking on Iron Surfaces," ACS Catalysis **14**, 14652–14664 (2024).

[3] S. Chen, J. Jelic, D. Rein, S. Najafishirtari, F. P. Schmidt, F. Girgsdies, L. Kang, A. Wandzilak, A. Rabe, D. E. Doronkin, J. Wang, K. Friedel Ortega, S. DeBeer, J. D. Grunwaldt, R. Schlögl, T. Lunkenbein, F. Studt, and M. Behrens, "Highly loaded bimetallic iron-cobalt catalysts for hydrogen release from ammonia," Nature Communications 2024 15:1 **15**, 1–11 (2024).

[4] M. Purcel, A. S. Müller, P. Diehl, K. Kappis, A. Trunschke, and M. Muhler, "Long-term stability of ammonia decomposition over nickel-based catalysts," Energy Technology **13**, 2400678 (2025).
